# The Measurement Properties and Applications of WHODAS 2.0 in Multiple Sclerosis: A Systematic Review

**DOI:** 10.3390/medsci14050542

**Published:** 2026-09-02

**Authors:** Amelia Rizzo, Maria Grazia Maggio, Andrea Calderone, Federica Impellizzeri, Giulia Pistorino, Claudia Pitrone, Aldo Sitibondo, Fabrizio Meus, Rita Mazziotti, Raffaele Migliorini, Diego Burzomati, Rocco Salvatore Calabrò

**Affiliations:** 1 Medico-Legal Centre of Messina, National Institute of Social Welfare (INPS), Via Giuseppe Garibaldi 124, 98122 Messina, Italy; amrizzo@unime.it (A.R.); claudia.pitrone01@inps.it (C.P.); aldo.sitibondo@inps.it (A.S.); diego.burzomati@inps.it (D.B.); 2 IRCCS Centro Neurolesi Bonino Pulejo, S.S. 113, Via Palermo, Contrada Casazza, 98124 Messina, Italy; mariagrazia.maggio@irccsme.it (M.G.M.); roccos.calabro@irccsme.it (R.S.C.); 3 Department of Brain and Behavioral Sciences, University of Pavia, Via Agostino Bassi 21, 27100 Pavia, Italy; giulia.pistorino01@universitadipavia.it; 4 Medico-Legal Centre of Genova, National Institute of Social Welfare (INPS), Piazza della Vittoria 6R, 16121 Genova, Italy; fabrizio.meus@inps.it; 5 Medico-Legal General Coordination, National Institute of Social Welfare (INPS), Via Ciro il Grande, 21, 00144 Rome, Italy; rita.mazziotti@inps.it (R.M.); raffaele.migliorini@inps.it (R.M.)

**Keywords:** multiple sclerosis, World Health Organization Disability Assessment Schedule, WHODAS 2.0, disability, quality of life, patient-reported outcomes, functioning, rehabilitation

## Abstract

**Background/Objectives:** Multiple sclerosis (MS) affects motor, cognitive, psychological, and social functioning that impairment-based scales do not fully capture. This systematic review evaluated the measurement properties and applications of the World Health Organization Disability Assessment Schedule (WHODAS) 2.0 in adults with MS across research and clinical contexts. **Methods:** PubMed/MEDLINE, Embase, Scopus, Web of Science, and the Cochrane Library were searched from inception to 25 May 2026. Eligible peer-reviewed studies reported extractable WHODAS data in adults with MS. Evidence was organized into measurement, clinical-association, participation/employment, intervention, digital-monitoring, and longitudinal streams. Design-appropriate critical-appraisal tools and outcome-specific certainty assessments were applied; quantitative pooling was assessed but was not justified. **Results:** Of the 677 records identified, 28 reports were included. Five reports evaluated measurement properties; moderate-certainty evidence supported reliability and construct validity, although precision differed among 12-, 32-, and 36-item forms. Associations with quality of life and clinical severity were supported by low-certainty evidence. Evidence concerning fatigue, mood, cognition, participation, and employment was of low or very low certainty because most studies were cross-sectional, used heterogeneous metrics, or involved overlapping cohorts. Intervention evidence was of very low to low certainty: WHODAS was secondary, and interventions, time points, and scoring methods were not comparable. Eight reports arose from one overlapping research program. **Conclusions:** WHODAS 2.0 provides a multidimensional patient-reported account of functioning in MS and may complement, but not replace, the Expanded Disability Status Scale. Evidence supports measurement use more strongly than prognostic, treatment-response, or benefit-determination claims. Standardized version selection, scoring, domain reporting, and prespecified longitudinal endpoints are required.

## 1. Introduction

Multiple sclerosis (MS) is a chronic immune-mediated disease of the central nervous system and a leading cause of non-traumatic neurological disability in young and middle-aged adults, with an increasing global prevalence and a substantial individual and societal burden [1,2]. Diagnosis is supported by contemporary criteria that integrate clinical, radiological, and laboratory evidence of dissemination in space and time [3], whereas the clinical course may include relapsing–remitting, secondary progressive, and primary progressive patterns with different implications for monitoring, prognosis, treatment response, and outcome assessment [4]. Because MS commonly begins during early adulthood and may persist for decades, disability develops within family, occupational, and community contexts rather than within neurological examination alone. A clinically useful assessment of disability, therefore, needs to capture how impairments are translated into daily functioning, perceived limitations, and restrictions in participation.

Disability assessment is increasingly framed internationally through the International Classification of Functioning, Disability and Health (ICF), which distinguishes body impairment from limitations in activities and restrictions on participation. In Italy, Law No. 227/2021 and Ministerial Decree No. 94/2024 provide a national example of this broader transition toward an ICF-oriented, multidimensional approach to disability assessment; however, this jurisdiction-specific context does not establish the clinical validity, prognostic performance, or eligibility-determination utility of WHODAS 2.0 in MS. National systems may operationalize this framework differently; therefore, WHODAS scores should be interpreted as standardized descriptions of functioning rather than as direct determinants of legal status, entitlement, or organic impairment.

The Expanded Disability Status Scale (EDSS) remains the most established clinician-rated measure of neurological impairment and disability in MS [5]. Its long-standing role in trials and clinical cohorts reflects familiarity, historical comparability, and sensitivity to ambulatory milestones, yet its weighting toward walking capacity can underrepresent domains that matter to patients and it may need to be complemented by broader clinical, imaging, biomarker, and patient-reported outcome approaches when assessing disease progression and treatment response [6]. Contemporary outcome frameworks for relapsing–remitting MS increasingly emphasize health outcomes that are meaningful for both clinicians and patients, including functioning, symptoms, quality of life, work participation, and treatment impact [7]. The International Classification of Functioning, Disability and Health (ICF) provides a biopsychosocial framework in which disability emerges from interactions among health conditions, body functions and structures, activities, participation, environmental factors, and personal factors; recent MS-specific ICF work reinforces the relevance of multidimensional functioning beyond impairment severity alone [8].

The World Health Organization Disability Assessment Schedule (WHODAS 2.0) was developed by the World Health Organization as a generic, cross-condition instrument for measuring health and disability in a way that is conceptually aligned with the ICF [9]. It assesses cognition, mobility, self-care, getting along, life activities, and participation, using standardized scoring procedures that permit overall and domain-specific disability profiles. Its generic structure may support comparisons across neurological and non-neurological conditions, while its self-reported format may complement clinician-rated neurological scales by incorporating the patient’s experience of functioning [10]. This is particularly relevant in MS because major contributors to perceived disability and quality of life include domains that are not fully captured by ambulatory scales, such as cognitive impairment, mood symptoms, fatigue, and social or occupational participation [11,12,13].

The instrument’s generic scope creates both an opportunity and a methodological problem. A common ICF-aligned framework can connect clinical, rehabilitation, and participation research, but generic breadth does not ensure that every form has sufficient precision for individual MS care or that the same total score has the same meaning under different scoring algorithms. Moreover, a study may use WHODAS to validate another instrument, to describe a sample, to predict a non-WHODAS outcome, or to measure change. These uses require different comparators, risk-of-bias judgments, and certainty statements. A review centered on the act and outcome of measurement can preserve that distinction while still describing the full range of applications.

Psychological and rehabilitation interventions further illustrate the need for multidimensional outcomes. Cognitive behavioral therapy and exercise-based approaches address symptoms, self-management, activity, and participation, as well as physical performance [14,15,16]. WHODAS 2.0 may, therefore, be informative when functioning is a prespecified outcome, provided that its role and version are reported explicitly.

Employment is a salient participation outcome because fatigue, mood, disability, comorbidity, and workplace factors can influence job retention and work difficulty in MS [17,18,19,20]. This context supports examining WHODAS-based participation and work findings as a distinct evidence stream rather than treating them as quality-of-life evidence.

Despite this broader literature, the empirical use of WHODAS 2.0 in MS remains fragmented across psychometric validation studies, observational analyses, longitudinal cohorts, work and participation research, and intervention trials. Variation in WHODAS version, scoring method, comparator choice, quality-of-life instrument, and reporting of EDSS or psychological variables limits direct interpretation across studies. A systematic synthesis is, therefore, needed to clarify how WHODAS 2.0 has been applied in MS, how its scores relate to clinical and patient-centered outcomes, and where methodological gaps remain.

A central distinction is whether a study evaluates WHODAS itself or merely uses a WHODAS score while addressing another question. Reliability, dimensionality, and interpretability studies inform measurement performance; cross-sectional correlations inform construct relationships; trials inform a specific intervention contrast; and trajectory studies describe change under their own modeling assumptions. Treating these reports as one homogeneous body of evidence would obscure both the role of the instrument and the certainty attached to each claim. The present review, therefore, uses one eligibility framework but separates synthesis, appraisal, and certainty by measurement purpose.

Accordingly, this review focuses on the measurement outcome: how WHODAS 2.0 has been measured, scored, and used in adults with MS, and what inferences are supported within distinct study-design and outcome streams.

This systematic review aimed to synthesize evidence on the measurement properties of WHODAS 2.0 and its associations with clinical status and quality of life in multiple sclerosis, while also examining its applications in participation and employment research, intervention and responsiveness assessment, digital monitoring, and longitudinal or psychosocial investigations. Evidence within each domain was evaluated using appraisal tools and certainty-assessment methods appropriate to the underlying study design. Throughout the review, WHODAS 2.0 was conceptualized as a complementary measure of multidimensional functioning that captures aspects of disability not fully represented by impairment-focused assessments, rather than as a replacement for the Expanded Disability Status Scale.

## 2. Materials and Methods

### 2.1. Study Design and Registration

This systematic review was conducted and reported in accordance with the Preferred Reporting Items for Systematic Reviews and Meta-Analyses (PRISMA) 2020 statement, with search reporting informed by the PRISMA extension for Searching (PRISMA-S) and protocol registration aligned with the International Prospective Register of Systematic Reviews (PROSPERO) guidance [21,22,23]. The protocol was prospectively registered on 23 May 2026 (CRD420261403510). Because the corpus included measurement, observational, longitudinal, and intervention evidence, the review question was structured with a Population–Concept–Context (PCC) framework and review-defined evidence streams appropriate to the heterogeneous evidence base and research questions (Supplementary Table S1).

### 2.2. Eligibility Criteria, PCC Framework, and Review Questions

The PCC framework comprised adults with MS (Population), measurement or application of WHODAS 2.0/WHO-DAS II (Concept), and any clinical, community, rehabilitation, occupational, digital, or longitudinal research setting (Context). The primary question was how WHODAS was implemented and what measurement inferences were supported. Secondary questions addressed design-specific associations or changes within six review-defined evidence streams (Supplementary Table S2).

The six streams were defined as an organizational framework for the synthesis; this classification did not alter the registered population, target instrument, or substantive eligibility criteria. Measurement-property evidence included reliability, structural validity, construct validity, responsiveness, and interpretability. Clinical-association evidence included quality of life, EDSS, fatigue, mood, cognition, and motor performance. Participation/employment evidence included social participation, work difficulty, employment status, and work transition. Intervention evidence included randomized and non-randomized change or response analyses. Digital/remote evidence included sensor and remote-monitoring applications. Longitudinal/psychosocial evidence included disability trajectories, relapse profiles, and structural models. A report could inform more than one topic, but one primary stream was assigned for descriptive counts to avoid inflating the visual evidence map.

Eligible WHODAS implementations included 12-, 32-, and 36-item forms; self- or interviewer administration; simple, transformed, or Rasch scoring; total or domain scores; and use as a measurement target, primary outcome, predictor, correlate, secondary outcome, or contextual descriptor. A comparator was not required for psychometric or descriptive studies. Eligibility did not imply that these different uses represented a homogeneous estimand.

The Population criterion was intentionally broad enough to include relapsing–remitting, secondary progressive, primary progressive, and mixed MS samples, but the Population element did not erase clinical heterogeneity. Mixed-disease reports were eligible only when the MS subgroup was described and contributed extractable WHODAS-related information. The Concept element required empirical administration and reporting of WHODAS, not a mention in background or protocol text. The Context element admitted community, outpatient, rehabilitation, occupational, and remote settings because context can influence disability reporting. Case reports and very small descriptive series were excluded because they could not contribute stable measurement or association estimates. Companion reports were not automatically excluded: eligibility depended on whether the article added a distinct measurement property, outcome, follow-up, or analytic question.

#### 2.2.1. Inclusion Criteria

Original peer-reviewed articles published in English were eligible for inclusion when they reported empirical data on adults with MS and used WHODAS 2.0 as a measure of disability, functioning, participation, or related patient-centered outcomes. Eligible populations included participants with any MS phenotype, including relapsing–remitting, secondary progressive, primary progressive, or mixed MS samples, regardless of disease duration, disability severity, treatment status, rehabilitation exposure, or clinical setting. Studies conducted in outpatient clinics, hospital settings, rehabilitation units, community samples, occupational contexts, or digital intervention settings were considered eligible when the MS population and WHODAS-related data were clearly identifiable.

Studies including mixed neurological, psychiatric, musculoskeletal, or chronic disease populations were included only when data for participants with MS could be separately extracted or when the MS subgroup represented a clearly defined analytical component of the study. WHODAS 2.0 could be used as a primary or secondary outcome, descriptive disability measure, predictor, correlate, psychometric target, or component of a broader functioning assessment. Both the 12-item and 36-item versions were considered eligible, including self-administered and interviewer-administered formats, total scores, domain scores, simple or complex scoring procedures, and validated translated versions.

Eligible study designs included cross-sectional studies, cohort studies, longitudinal studies, psychometric validation studies, randomized controlled trials, non-randomized intervention studies, quasi-experimental studies, registry-based analyses, and secondary analyses of clinical cohorts or trials. Studies were retained when they reported original WHODAS-related data, including descriptive scores, associations with clinical or patient-reported variables, psychometric properties, group comparisons, longitudinal changes, or intervention-related outcomes relevant to disability, quality of life, EDSS, fatigue, cognition, psychological symptoms, participation, employment, or rehabilitation.

#### 2.2.2. Exclusion Criteria

Studies were excluded when they were not original peer-reviewed articles, were not in English, or did not report extractable WHODAS data in adults with MS. Reviews, editorials, letters, guidelines, chapters, conference abstracts, proceedings, theses, dissertations, trial registrations, and protocols without results were excluded. Gray literature was not searched because eligibility was restricted a priori to peer-reviewed full articles; the resulting risk of publication and language bias was considered in certainty judgments.

Case reports and very small descriptive case series were excluded because they did not provide sufficient analytical evidence for the review question. Studies were also excluded when MS participants were combined with other populations without separate MS-specific data, when WHODAS 2.0 was mentioned only in the background, rationale, search strategy, or planned methods without reporting empirical results, or when disability/functioning was assessed exclusively with instruments other than WHODAS 2.0. Studies focused only on pediatric MS, clinically isolated syndrome, radiologically isolated syndrome, neuromyelitis optica spectrum disorder, or other demyelinating diseases were excluded unless adult MS-specific WHODAS data were separately available. Duplicate or overlapping publications from the same cohort were managed by retaining the most complete report, while companion articles were considered only when they provided distinct WHODAS-related outcomes, follow-up data, intervention results, or psychometric information.

### 2.3. Information Sources and Search Strategy

PubMed/MEDLINE, Embase, Scopus, Web of Science, and the Cochrane Library were searched from database inception to 25 May 2026. The strategies combined MS terms with WHODAS/WHO-DAS names and version variants. Exact database-specific search strings are reproduced verbatim in Supplementary Table S3B, while the corresponding field selections, search dates, limits, operator use, language handling, and retrieval counts are reported in Supplementary Table S3A. No controlled-vocabulary terms, proximity operators, date limits, or methodological filters were applied beyond the documented interface settings, and no unexecuted syntax was added retrospectively.

The strategy deliberately included historical spellings and punctuation because early articles used WHO-DAS II or WHODAS-II, whereas later reports used WHODAS 2.0 and version-specific labels. MS phenotype terms were retained to capture records indexed around relapsing–remitting or progressive disease rather than the generic phrase alone. Broader disability, functioning, quality-of-life, employment, or rehabilitation terms were not required, because adding an outcome block could reduce sensitivity for psychometric or secondary-use reports. Conversely, the target-instrument block was required because the review did not seek every disability instrument used in MS. This two-concept structure explains the high Scopus yield and the need for manual screening, but it also makes the instrument criterion transparent and reproducible.

The exact database-specific search strings are reproduced verbatim in Supplementary Table S3B, while Supplementary Table S3A records the corresponding database fields, search date, limits, operators, and retrieved counts. The searches retrieved 27 PubMed, 59 Embase, 548 Scopus, 28 Web of Science, and 15 Cochrane records (total 677). The results were exported, electronically deduplicated, and manually checked for residual duplicates.

Search reproducibility was documented through the verbatim database-specific strings and corresponding execution metadata reported in Supplementary Table S3A,B. English language was applied as an eligibility criterion rather than as a database filter. Citation tracking and gray-literature searching were not additional sources of eligible records. This narrower publication-type scope improves the consistency of the included corpus but may preferentially retain positive or fully reported findings.

### 2.4. Study Selection Process

Study selection was performed in two sequential stages by two independent reviewers, A.R. and M.G.M. Before formal screening, the reviewers completed a calibration exercise on a pilot set of records to harmonize the interpretation of WHODAS terminology, MS-specific extractability, eligible study designs, and mixed-sample reporting. First, titles and abstracts were screened independently against the eligibility criteria. Title and abstract exclusions were recorded separately in the screening database to distinguish clearly irrelevant records from records excluded after abstract-level assessment. Records judged potentially eligible by either reviewer were advanced to full-text assessment to minimize erroneous exclusion. Second, the same two reviewers independently assessed full-text reports. Disagreements at either stage were resolved through discussion, and unresolved disagreements were adjudicated by a third reviewer, R.S.C. Reasons for exclusion at the full-text stage were recorded using mutually exclusive categories, including wrong population, no extractable WHODAS 2.0 data, ineligible publication type or design, non-extractable MS subgroup, duplicate or overlapping report, adjacent disability instrument, or insufficient empirical data. Inter-rater agreement between A.R. and M.G.M. was substantial, with Cohen’s κ = 0.758 for title and abstract screening and κ = 0.764 for full-text eligibility assessment. Multiple reports derived from the same cohort were compared before synthesis; companion articles were retained only when they provided distinct WHODAS-related information, and overlap was recorded to avoid double counting. The operational workflow is detailed in Supplementary Table S4.

Screening decisions were documented at the report level so that multiple papers from one program could be distinguished from duplicate database records. A duplicate was removed before screening when bibliographic information identified the same report. A companion article was evaluated in full text because it could contain a different outcome or follow-up. Full-text categories were made mutually exclusive by assigning the first decisive reason in a fixed operational hierarchy: target instrument, population, publication type, overlap, adjacent instrument, and empirical sufficiency. This approach preserved a transparent total of twenty full-text exclusions while allowing overlap among retained reports to be handled during synthesis. No potentially eligible full text was unavailable after retrieval attempts. Full-text exclusion categories and counts are reported in Supplementary Table S5.

### 2.5. Data Extraction and Data Items

Two reviewers (A.R. and M.G.M.) independently extracted study, population, design, WHODAS version, administration, scoring, outcome timing, numerical results, and contextual variables. In response to heterogeneity of purpose, extraction also coded WHODAS as a measurement target, central outcome/predictor, or secondary/supportive measure; recorded whether it was primary or secondary when authors specified this; and assigned reports to a cohort family. Potential or probable overlap was flagged before any sample or evidence count was interpreted. Missing details were recorded as not reported and were not inferred. The complete extraction structure is provided in Supplementary Table S6. Disagreements were resolved by discussion; unresolved disagreements were adjudicated by R.S.C.

Numerical extraction followed a source hierarchy established before comparison of findings. Values in primary tables were preferred to figures and narrative summaries when discrepancies occurred; adjusted estimates were preferred for etiological or prognostic interpretation, whereas unadjusted estimates were retained when they were the only source-reported result. Confidence intervals, standard errors, *p* values, and model-fit statistics were recorded with the associated outcome definition and time point. For trials, extraction distinguished randomized sample, analyzed sample, within-group change, and between-group contrast. For psychometric reports, the unit of evidence was the measurement property rather than a generic study result. No effect size was calculated from incomplete data, and no unstated WHODAS scoring algorithm was imputed.

Cohort overlap was evaluated from program names, author groups, recruitment sites, enrolment periods, eligibility criteria, sample descriptions, and reported participant numbers. Reports were classified as clearly independent, having probable overlap, having possible overlap, or a companion report within a named program. The eight reports from the Trajectories of Outcome in Neurological Conditions in Multiple Sclerosis (TONiC-MS) program were treated as one program family despite their different analytic subsets and sample sizes. The Gandy intervention reports, two Italian Multiple Sclerosis Questionnaire for Job Difficulties (MSQ-Job) reports, and two Magistrale reports were also flagged as having probable or possible overlap. Distinct reports were retained when they answered different questions, but their sample sizes were not summed to represent unique participants and their consistency was not treated as independent replication.

Analytic role was coded independently of study design. A measurement target meant that the report directly evaluated a WHODAS property. A central outcome or predictor meant that a WHODAS score defined the main disability endpoint, response, trajectory, or principal explanatory variable. A secondary/supportive role meant that WHODAS provided context, convergent evidence, covariate information, or one outcome among several when the primary question concerned another construct. When authors did not explicitly label an endpoint as primary or secondary, classification was based on the stated objective, analysis plan, and prominence of the WHODAS result. This coding was used for interpretation and evidence mapping, not as a post hoc eligibility threshold.

### 2.6. Risk-of-Bias Assessment

Risk of bias or methodological quality was assessed independently by A.R. and M.G.M. using design-appropriate tools; unresolved disagreements were adjudicated by R.S.C. Randomized trials used the revised Cochrane Risk of Bias tool for randomized trials (RoB 2) [24]; the non-randomized intervention used the Risk Of Bias In Non-randomized Studies of Interventions (ROBINS-I) [25]; measurement-property studies used the COnsensus-based Standards for the selection of health Measurement INstruments (COSMIN) Risk of Bias framework [26]; analytical cross-sectional studies used the Joanna Briggs Institute (JBI) checklist [27]; and prognostic-factor or trajectory analyses used the Quality In Prognosis Studies (QUIPS) tool [28]. Domain judgments were resolved by consensus, were not converted to numerical quality scores, and informed stream-specific interpretation. Tool allocation and study-level synthesis concerns are reported in Supplementary Tables S7 and S8A; the retained cross-report domain matrix is reproduced in Table S8B.

Tool choice followed the question actually answered by each report. RoB 2 judgments were outcome-specific for the WHODAS result rather than inherited from a trial’s primary endpoint. ROBINS-I was restricted to the single non-randomized intervention and was not imposed as the formal appraisal tool on cross-sectional or psychometric designs. COSMIN judgments considered whether sample, administration, statistical analysis, and reporting were adequate for each claimed measurement property. JBI appraisal emphasized participant selection, valid exposure/outcome measurement, confounding, and analysis. QUIPS emphasized study participation, attrition, measurement of the prognostic factor and outcome, confounding, and model reporting. As these tools use different domains and labels, judgments were not combined into a global score or ranked across designs. The original cross-report, ROBINS-I-aligned domain matrix was retained solely as a descriptive visualization of recurrent limitations among non-randomized reports; it did not replace the formal design-specific appraisals or determine certainty ratings.

### 2.7. Certainty of Evidence

Certainty was assessed separately for measurement properties, quality of life, clinical severity/motor–cognitive functioning, psychological correlates, participation/employment, intervention response, and longitudinal trajectories [29,30]. The Grading of Recommendations Assessment, Development and Evaluation (GRADE) domains were applied to association and intervention questions, whereas certainty in measurement properties was integrated with COSMIN appraisal. Overlapping reports contributed distinct findings but did not increase certainty as if they were independent cohorts. The framework is specified in Supplementary Table S9.

For each stream, certainty judgments considered the limitations of the relevant designs rather than the prestige or size of an individual report. Inconsistency referred to unexplained differences in direction or magnitude among studies addressing a comparable construct; heterogeneity alone was not labeled inconsistency when studies asked different questions. Indirectness included mixed-disease validation, a secondary WHODAS endpoint, or a work-specific construct used as a proxy for broader participation. Imprecision considered confidence intervals, sample size, event count, and stability of model estimates. Publication bias was considered plausible because the corpus excluded gray literature and contained small streams. Large samples did not justify upgrading when they arose from overlapping program data or when residual confounding remained.

### 2.8. Data Synthesis

The primary synthesis followed Synthesis Without Meta-analysis (SWiM) guidance [31] and preserved the six review-defined streams. Before deciding against meta-analysis, two reviewers audited every stream for at least two independent reports with the same construct, compatible WHODAS form/scoring, comparable time points, and a common estimand. Although several streams contained multiple numerical results, no candidate pool satisfied all criteria. Converting raw WHODAS scores to a common range would not harmonize correlations, regression coefficients, group contrasts, change scores, or trajectory parameters, and standardization would not resolve cohort overlap. Consequently, zero quantitative pools were undertaken; the synthesis rules, reporting architecture, certainty summaries, study-level matrices, and feasibility audit are reported in Supplementary Tables S10–S15.

Within each narrative group, the synthesis compared the direction of association or effect, approximate magnitude, precision, adjustment, outcome status, and appraisal concerns. Studies were not assigned vote-counting weights according to statistical significance. Large longitudinal analyses were emphasized for temporal description but were explicitly linked to their cohort family. Small cross-sectional studies were retained when they addressed a distinct domain, but their results were not used to imply prevalence or causal mechanism. Evidence gaps were mapped for progressive MS, domain-level scoring, independent replication, minimal important change, and prespecified WHODAS endpoints. This hierarchy allowed numerical signals to remain visible without manufacturing comparability.

The feasibility audit did not equate the presence of two numerical values with poolability. Reliability coefficients, factor-model fit, correlations, adjusted regression coefficients, odds ratios, between-group differences, standardized mean differences, and latent-trajectory parameters answer different questions. Potential conversion to a 0–100 WHODAS scale was considered only for raw means or changes, but comparable variance and time-point data were generally unavailable and intervention estimands remained different. Fisher transformations of correlations were not used because the correlated constructs and cohorts differed. No effect was excluded from the narrative solely because it could not be standardized.

Supplementary Table S16 reports the detailed quantitative findings, effect-size signals, and study-specific interpretive limitations transferred from the main manuscript.

## 3. Results

### 3.1. Study Selection and PRISMA Flow

The five databases yielded 677 records: PubMed 27, Web of Science 28, Cochrane Library 15, Embase 59, and Scopus 548. After the removal of 100 duplicates, 577 records underwent title/abstract screening and 529 were excluded. Forty-eight full texts were assessed; all were retrieved. The reconciled PRISMA counts are reported in Supplementary Table S11.

**Figure 1:**
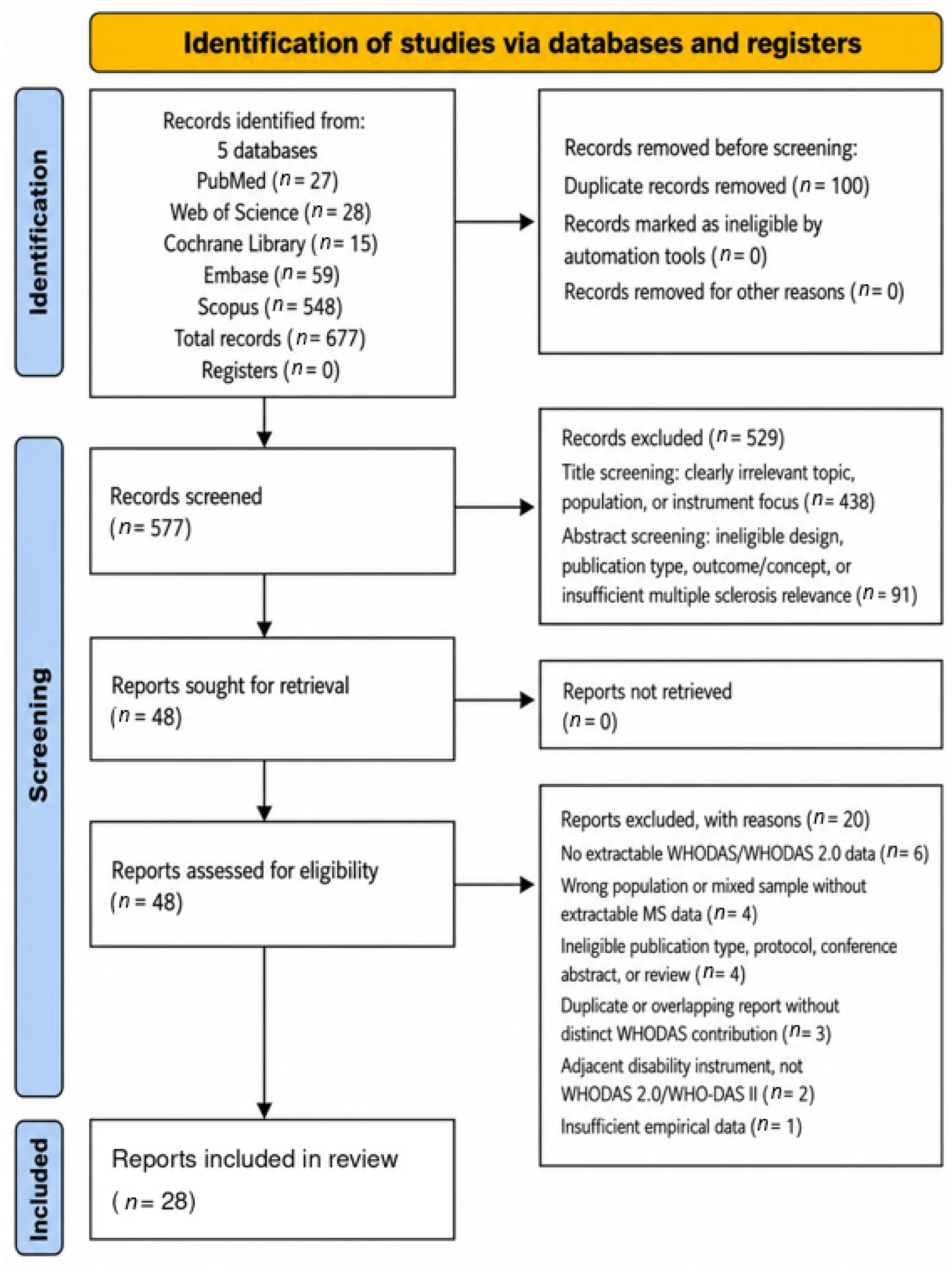
PRISMA 2020 flow diagram of study selection. The flow diagram summarizes the identification, screening, eligibility assessment, and inclusion process for reports evaluating WHODAS 2.0 or WHO-DAS II in adults with multiple sclerosis. Database searches identified 677 records from PubMed, Web of Science, Cochrane Library, Embase, and Scopus. After duplicate removal and title/abstract screening, 48 reports were assessed for eligibility and 28 reports were included in the narrative synthesis.

Twenty full-text reports were excluded for the following reasons: no extractable WHODAS data (*n* = 6), wrong population or non-extractable MS subgroup (*n* = 4), ineligible publication type (*n* = 4), duplicate/overlapping report without a distinct contribution (*n* = 3), adjacent disability instrument (*n* = 2), and insufficient empirical data (*n* = 1). Twenty-eight reports were included [32,33,34,35,36,37,38,39,40,41,42,43,44,45,46,47,48,49,50,51,52,53,54,55,56,57,58,59]. Figure 1 provides the complete flow; the exclusion categories and reconciled counts are detailed in Supplementary Tables S5 and S11.

The included corpus spanned publication years 2007–2026 and ranged from 56 randomized participants in a small balance trial to more than 5000 participants in several TONiC-MS analyses. Three reports were randomized trials, one was a non-randomized intervention, five were primarily measurement-property studies, six were classified as prognostic or trajectory analyses, and thirteen were analytical cross-sectional reports. These design counts describe the appraisal allocation and should not be interpreted as mutually independent samples. The single mixed-disease validation report contributed an MS subgroup; studies focused on occupation or remote monitoring often treated WHODAS as a supporting variable rather than the main outcome.

### 3.2. Risk of Bias, Certainty, and Interpretive Confidence

**Figure 2:**
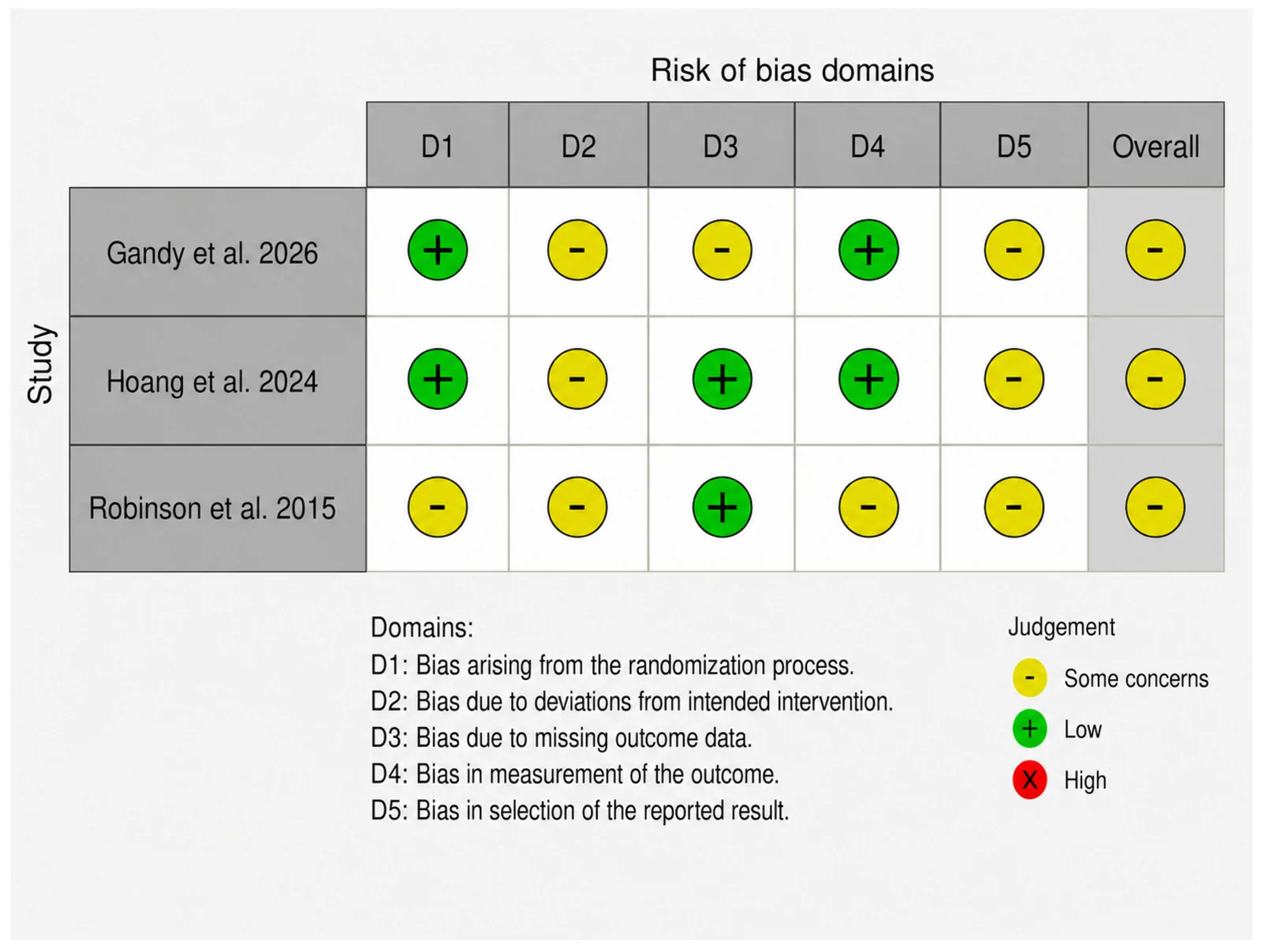
Risk-of-bias assessment of randomized controlled trials using RoB 2 [36,38,46]. Risk of bias in randomized controlled trials was assessed using the revised Cochrane Risk of Bias tool for randomized trials.

The three randomized trials were appraised with RoB 2 [36,38,46]. Each had some concerns, principally because WHODAS was patient-reported, was secondary in two trials, or had incomplete outcome reporting. These trials addressed dissimilar interventions and estimands and were not pooled. Figure 2 presents domain-level judgments.

**Figure 3:**
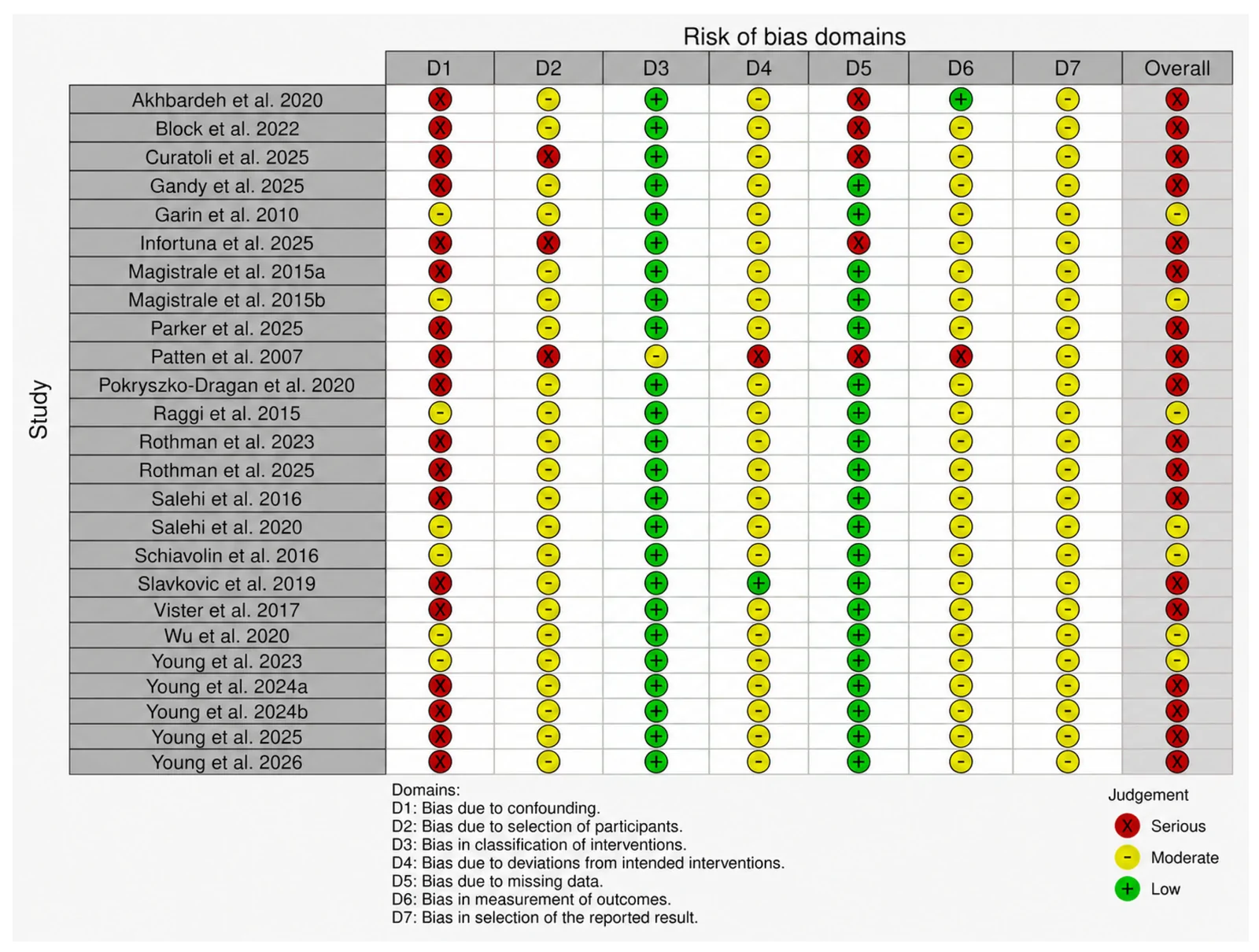
Cross-report risk-of-bias overview for non-randomized included reports [32,33,34,35,37,39,40,41,42,43,44,45,47,48,49,50,51,52,53,54,55,56,57,58,59]. This descriptive visualization does not replace the design-specific appraisals in Supplementary Tables S7 and S8A, is reproduced for transparency in Supplementary Table S8B, and was not used to rank evidence across tools.

The remaining reports were assigned according to design and analytic purpose: one non-randomized intervention to ROBINS-I, five measurement-property reports to COSMIN, six prognostic/trajectory reports to QUIPS, and thirteen analytical cross-sectional reports to the JBI checklist. Supplementary Tables S7 and S8A report this formal allocation and the synthesis-relevant concerns. Figure 3 provides the retained cross-report domain overview for the 25 non-randomized reports; the corresponding domain matrix is reproduced in Supplementary Table S8B.

Appraisal identified different recurrent concerns across streams. The randomized trials generally raised some concerns rather than a uniform high-risk judgment. The non-randomized depression-management report was more vulnerable to confounding and selection. Measurement reports varied in sample definition, missing-data handling, subscale fit, and cross-cultural factor structure, although the total-score evidence was comparatively coherent. Cross-sectional reports commonly lacked temporal ordering and could not fully separate neurological severity, fatigue, mood, and perceived disability. Prognostic and trajectory reports benefited from larger samples or repeated data but were limited by attrition, model selection, residual confounding, and cohort overlap. These design-specific concerns were carried into certainty judgments rather than summarized as one overall percentage.

Within the five measurement-property reports, certainty was moderate for total-score reliability and construct validity, but low for structural validity/dimensionality and for responsiveness or individual interpretability because the directly informative evidence was sparse and form-specific [37,41,50,54,55]. Quality-of-life and clinical-severity associations were of low certainty. Psychological, cognitive, participation, and employment findings were of low to very low certainty because most were cross-sectional, incompletely adjusted, or based on overlapping cohorts. Intervention evidence was of very low to low certainty because WHODAS was secondary in several studies and interventions, forms, metrics, and time points differed [35,36,38,43,46]. Longitudinal evidence was of low certainty and was not strengthened by counting eight TONiC-MS reports as independent cohorts [42,47,48,55,56,57,58,59]. Property-specific judgments are reported in Supplementary Table S12A,B.

No certainty label was extrapolated from one stream to another. Moderate certainty for measurement properties means reasonable confidence that selected WHODAS total-score implementations measure functioning consistently in MS; it does not demonstrate diagnostic accuracy, a minimal clinically important difference, predictive validity, or effectiveness as an intervention endpoint. Low certainty for longitudinal evidence reflects confidence in descriptive associations and trajectories, not proof that WHODAS forecasts an individual outcome. Very-low- to low-certainty intervention evidence means that the true treatment-related change may differ substantially from reported estimates.

### 3.3. WHODAS 2.0 Use Across Study Designs and Outcome Domains

The 28 reports were heterogeneous in design and in the analytic importance of WHODAS. Five directly evaluated measurement properties, eleven used WHODAS as a central outcome or predictor, and twelve used it as a secondary or supportive measure. Table 1 retains the key study characteristics and role needed for interpretation; study-level details are provided in Supplementary Tables S13 and S14.

The distinction between central and supportive use materially changed interpretation. In the five measurement reports, WHODAS properties were the object of analysis. In central-outcome studies, the score defined perceived disability, treatment response, a trajectory, or a principal predictor. In supportive studies, WHODAS was one variable in a broader falls, employment, psychosocial, occupational, or rehabilitation analysis. A significant result from the latter group cannot establish broad measurement performance, and the absence of a WHODAS effect may reflect limited power or secondary reporting. This role classification is, therefore, reported beside design and form rather than being treated as a descriptive afterthought.

Six reports predominantly used a 12-item form, nine used a 36-item/WHO-DAS II form, eight used a 32-item or Rasch-based implementation, and five did not report the form sufficiently for confident classification. These counts describe dominant implementation and do not imply interchangeability. In particular, a validation target, a primary disability endpoint, and a peripheral covariate provide different types of evidence.

Administration and scoring detail also varied within these categories. Some 36-item studies reported simple sums or transformed 0–100 scores, whereas others used item response theory (IRT) or Rasch estimates. The 32-item implementation generally removed work-specific items to permit a common score among people who were and were not employed. Short-form studies used ranges of 0–48 or transformed 0–100 scores. Several reports provided total scores without enough information to determine whether the simple or complex World Health Organization (WHO) algorithm was used. Domain-level analysis was inconsistent, and treatment of work/school items was not always described. These differences affect both the meaning of a one-point change and the feasibility of direct comparison.

Reports were, therefore, synthesized within measurement, clinical-association, participation/employment, intervention/response, digital/remote-monitoring, and longitudinal/psychosocial streams. Table 2 summarizes these streams, their WHODAS roles, and certainty; detailed scoring and cohort-overlap fields are in the Supplementary Material.

**Figure 4:**
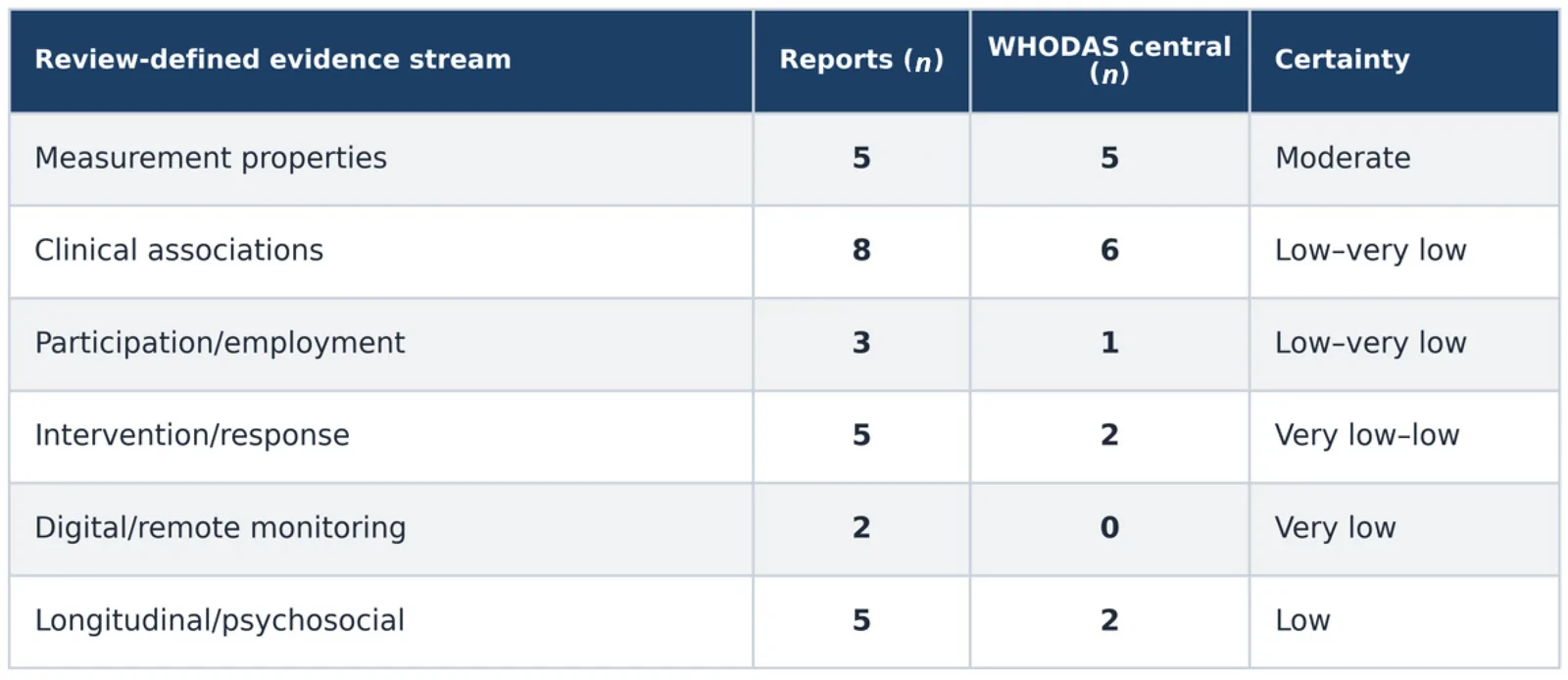
Evidence architecture of WHODAS 2.0 applications in MS. The matrix reports the number of included reports and the subset in which WHODAS was a measurement target, primary outcome, or principal predictor. Certainty is outcome-stream specific. Counts are descriptive and are not independent-cohort weights. The headline measurement-stream rating is disaggregated by property in Supplementary Table S12B.

Figure 4 displays the resulting evidence architecture. Counts refer to reports, not independent cohorts, and the number of reports in which WHODAS was central is shown separately. Eight reports derived from TONiC-MS; the Gandy, MSQ-Job, and Magistrale publications also contained probable or possible overlap. Thus, larger cells do not necessarily represent more independent evidence.

**Figure 5:**
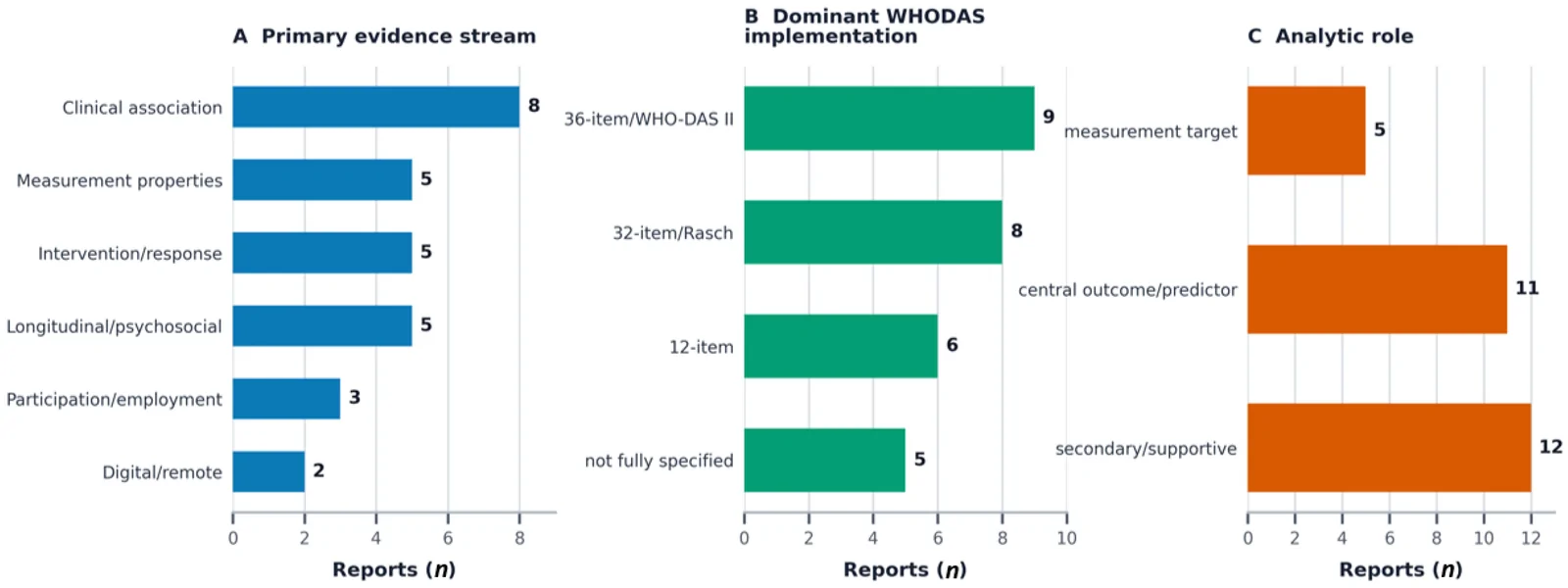
Distribution of WHODAS 2.0 evidence across the 28 included reports. (**A**) Distribution of reports according to the primary evidence stream, including clinical associations, measurement properties, intervention/response, longitudinal/psychosocial outcomes, participation/employment, and digital/remote applications. (**B**) Distribution according to the dominant WHODAS implementation, including the 36-item/WHODAS II, 32-item/Rasch-based, and 12-item versions, as well as reports in which the version was not fully specified. (**C**) Distribution according to the analytic role of WHODAS, categorized as measurement target, central outcome/predictor, or secondary/supportive measure. The overlap note identifies shared program data and related report clusters; counts must not be interpreted as numbers of independent cohorts.

Across the primary streams, clinical-association reports were most numerous (*n* = 8), followed by measurement, intervention/response, and longitudinal/psychosocial reports (*n* = 5 each), participation/employment reports (*n* = 3), and digital/remote reports (*n* = 2). WHODAS was central in all five measurement reports, six of eight clinical association reports, two of five longitudinal/psychosocial reports, two of five intervention reports, and one of three participation/employment reports; it was not central in either digital/remote report. This distribution explains why the evidence map separates report count from analytic centrality. Figure 5 complements the matrix by displaying primary stream, dominant WHODAS implementation, and analytic role while retaining the overlap safeguard.

### 3.4. Measurement Properties and Clinical Anchoring of WHODAS 2.0

Five reports directly evaluated measurement properties [37,41,50,54,55]. Garin et al. included 1119 participants across chronic diseases, with an extractable MS subgroup of 100, and, therefore, provided indirect MS evidence [37]. MS-specific studies supported total-scale reliability and construct validity, but also identified limitations in some subscales and in individual interpretation of the 12-item form [41,50,54,55]. The largest study concluded that 36- and 32-item Rasch estimates could support individual and group use, whereas the 12-item form supported group-level use [55]. These differences preclude treating forms and scores as interchangeable.

The quantitative measurement signals were not uniform. Magistrale et al. reported strong total-scale internal consistency and Rasch reliability, while noting unresolved subscale behavior [41]. The Persian adaptation reported domain intraclass correlations of 0.82–0.99 and convergent relationships with Multiple Sclerosis Quality of Life-54 (MSQoL-54), but sample and factor-analytic reporting warranted caution [50]. Wu et al. supported a two-factor solution for the 12-item form, with total α = 0.90 and acceptable modified confirmatory fit [54]. Young et al. provided transformation tables, smallest-detectable-difference estimates, longitudinal stability, and differential-item-functioning analyses [55]. These complementary properties support moderate certainty for measurement use, not moderate certainty for every clinical application.

Known-group and external-anchor findings added context but did not remove the form-specific limitations. Garin et al. observed gradients across chronic-disease severity and high reliability, yet their MS contribution came from only one subgroup within a heterogeneous sample [37]. Magistrale et al. compared 36- and 32-item scoring and showed that removing work items could improve applicability, while some domains retained floor effects or fit problems [41]. The Persian study supported translation validity and test–retest stability but yielded a seven-factor solution that was not directly comparable with the two-factor short-form result [50,54]. The TONiC-MS measurement report offered the broadest individual-versus-group interpretation, but companion articles using those transformations did not constitute new validation samples [55]. Detailed report-level quantitative findings, effect-size signals, and study-specific interpretive limitations are provided in Supplementary Table S16.

Clinical associations were consistent in direction but not in estimand. Sensor features correlated strongly with WHODAS (r_s_ = 0.82), and EDSS, anxiety, depression, fatigue, cognition, dexterity, and walking performance were associated with disability in separate models [32,34,40,52]. In remote monitoring, WHODAS contributed to a falls model (odds ratio [OR] 1.16, 95% confidence interval [CI] 1.08–1.25) but was one component of a broader predictor set [33]. These findings support convergent association, not prognostic validation.

More detailed analyses reinforced the multidimensional interpretation. Curatoli et al. reported independent coefficients for EDSS, state anxiety, and depressive symptoms in a model explaining 70.5% of WHODAS variance [34]. Infortuna et al. found that anxiety and hyperthymic temperament were associated with disability after adjustment [39]. Magistrale et al. reported a larger contribution from fatigue than from EDSS to total self-rated disability [40]. Pokryszko-Dragan et al. linked depression and mobility complaints with participation-related burden [44], and Slavkovic et al. identified cognitive, upper-limb, and walking contributions [52]. The diversity of predictors explains why one summary coefficient would be clinically ambiguous.

Quality-of-life associations were also observational. Total WHODAS scores were negatively correlated with MSQoL-54 scores (r = −0.71) in one study [49], while other reports used different quality-of-life instruments, structural models, or overlapping TONiC-MS data [42,47,48]. Directional consistency, therefore, did not justify pooling or causal interpretation.

Psychological findings showed a similar pattern. Anxiety, depression, fatigue, temperament, locus of control, and self-efficacy were examined with different instruments and statistical models [34,39,40,42,47,48,53,56]. Depressed TONiC-MS participants had higher mean WHODAS32 scores than those without depression, but that analysis shared a program with other longitudinal and psychosocial reports [56]. Vister et al. reported a correlation of 0.57 between fatigue and WHODAS and worse functioning among participants with low walking activity [53]. These signals support the proposition that self-reported disability reflects non-motor burden, while the cross-sectional designs prevent conclusions about whether symptoms precede, follow, or mutually reinforce disability.

### 3.5. Patient-Centered Functioning, Participation, Work, and Rehabilitation Outcomes

The narrative synthesis showed a coherent relationship between WHODAS and patient-centered outcomes. Quality-of-life evidence indicated that higher WHODAS disability was generally associated with lower quality of life or worse health status. This pattern was evident in cross-sectional quality-of-life analyses and in large TONiC-MS modeling, where fatigue, comorbidity burden, lower self-efficacy, and worse health status were associated with higher WHODAS disability [42,49]. Psychosocial modeling further positioned WHODAS as a functioning endpoint within pathways involving locus of control, self-efficacy, and quality of life [47,48]. These results support the use of WHODAS when the outcome of interest is not only impairment severity but also perceived functioning and life impact.

The large TONiC-MS program broadened the range of applications but also concentrated the evidence. Parker et al. modeled repeated WHODAS32 estimates in 5210 participants and reported associations with fatigue, comorbidity, and self-efficacy [42]. Rothman et al. used related program data to examine locus-of-control and self-efficacy pathways [47,48]. Young et al. examined measurement, depression, relapse trajectories, functioning near diagnosis, and employment using overlapping program subsets [55,56,57,58,59]. Each analysis addressed a distinct question; nevertheless, concordant directions across these articles cannot be interpreted as eight independent confirmations.

Psychological and fatigue-related evidence also converged across studies. Depression, anxiety, fatigue, temperament traits, mobility complaints, and walking activity were associated with WHODAS disability or participation-related functioning [39,44,53,56]. The TONiC-MS depression analysis reported higher WHODAS32 scores among participants with depression than among those without depression, with a moderate-to-large standardized difference and an odds ratio of 1.020 per WHODAS32 point [56]. The fatigue and falls cohort showed that fatigue severity was associated with worse WHODAS functioning, with r = 0.57, and that participants with high fatigue or low walking activity had higher WHODAS scores [53]. These findings were consistent in direction, but the synthesis did not treat them as causal because fatigue, mood, participation, and perceived disability may influence each other over time.

Participation and work-related evidence extended WHODAS into role functioning. Social participation research showed that WHODAS disability was related to depressive symptoms and mobility complaints [44]. Occupational studies showed strong associations between WHODAS and MS-specific job-difficulty measures, including a correlation of 0.761 between MSQ-Job total score and WHODAS summary disability [45]. A related cut-off study used WHODAS and MSQoL-54 clusters to define clinically meaningful MSQ-Job thresholds, with area-under-the-curve estimates above 0.84 [51]. Longitudinal employment evidence then linked WHODAS cognition and global WHODAS disability to adverse employment outcomes, including medical retirement [59]. These findings should be interpreted as convergent occupational evidence rather than as evidence from fully independent employment cohorts.

WHODAS was not equally positioned in these occupational analyses. In Raggi et al., it served as a convergent comparator during the development of a work-specific questionnaire [45]. In Schiavolin et al., WHODAS and quality-of-life clusters anchored a cut-off for that work instrument [51]. In Young et al., global disability and the cognition domain entered models of employment status and transition [59]. These uses show relevance to work participation, but none validates a stand-alone WHODAS threshold for employability, vocational allocation, or benefit eligibility.

Intervention evidence was heterogeneous. WHODAS was central in the Gandy response and randomized studies but secondary in the falls-prevention, depression-management, and balance-training studies [35,36,38,43,46]. The Gandy trial reported a small effect (Hedges g = 0.28); Hoang et al. reported no WHODAS difference (−0.4, 95% CI −3.5 to 2.6; *p* = 0.775); Patten et al. found no clear functional separation; and Robinson et al. reported adjusted short-term differences after two active balance interventions. These non-equivalent results provide very-low- to low-certainty evidence and do not establish general responsiveness.

The meta-analysis audit confirmed that the apparent multiplicity of intervention results did not yield one comparable set. Only four reports contained a group or change contrast, but the interventions addressed internet-delivered psychological care, unsupervised falls prevention, depression disease management, and short-term balance training. WHODAS forms, outcome status, analysis populations, follow-up intervals, and effect metrics differed. The longitudinal stream contained numerous numerical estimates, but most came from TONiC-MS and represented progression coefficients, group trajectories, structural paths, or employment models rather than repeated estimates of one effect. Similar incompatibility affected the quality-of-life, clinical, and measurement streams. The final number of feasible pools was, therefore, zero.

The source reports also differed in analysis population and interpretation. Gandy et al. defined disability response as at least 25% improvement among participants with mild baseline disability and found only modest baseline predictive discrimination [35]. The subsequent randomized trial evaluated a 10-week internet-delivered course with longer follow-up [36]. Hoang et al. randomized 461 participants, but falls—not WHODAS—were primary [38]. Patten et al. was a non-randomized study that administered the full functional module to a subset [43]. Robinson et al. randomized 56 participants and used complete-case analysis for a secondary short-term endpoint [46]. These differences remained after any conceivable rescaling.

Outcome direction also varied. The Gandy randomized trial favored an intervention with a small standardized effect, whereas the large step-exergame trial showed a WHODAS estimate close to the null [36,38]. The non-randomized depression-management study did not show a clear group difference in functioning [43]. The small balance trial reported larger adjusted short-term contrasts, but their stability is uncertain because of sample size, complete-case analysis, and the secondary status of WHODAS [46]. Pooling these estimates would obscure a clinically important fact: each result refers to a different intervention target, comparator, follow-up period, form, and estimand. The appropriate conclusion is that WHODAS has been feasible as an outcome in several settings, while general responsiveness remains unestablished.

## 4. Discussion

### 4.1. WHODAS 2.0 as a Bridge Between Neurological Disability and Lived Functioning

This review shows that WHODAS 2.0 has been used across measurement, clinical, occupational, intervention, digital, and longitudinal MS research. Its multidomain structure is consistent with current calls for outcome sets that include symptoms, functioning, and participation [60,61,62]. The strongest support concerns measurement properties; evidence for specific clinical, prognostic, or treatment-response uses is less certain.

This hierarchy resolves an apparent tension in the evidence. The broad recurrence of WHODAS across research contexts demonstrates feasibility and conceptual reach, but breadth of application is not equivalent to strength of validation. The instrument can be useful for describing a multidomain patient perspective while remaining insufficiently studied for a particular decision. Claims should, therefore, identify the form, score, role, and evidence stream rather than refer generically to the clinical value of WHODAS.

Digital and patient-reported outcomes can capture aspects of lived disability that performance measures alone may miss [63,64,65]. The included studies, nevertheless, show that a generic score does not remove the need to define the intended construct. WHODAS should be interpreted alongside EDSS, symptom scales, cognitive or motor testing, and MS-specific quality-of-life instruments when those constructs are relevant.

Fatigue, mood, cognition, and participation repeatedly correlated with WHODAS, consistent with continuing measurement challenges in these domains [66,67,68,69]. Because many measures were collected concurrently, the observed relationships may reflect shared patient-reported method variance or reciprocal effects. They should not be presented as evidence that WHODAS identifies causes of disability.

Participation and employment findings are clinically relevant but sparse. The Italian MSQ-Job reports were closely related, and the employment analysis arose from TONiC-MS. The evidence, therefore, supports WHODAS as a descriptor or correlate of role functioning, not as a validated tool for employment or benefit decisions.

Likewise, the intervention reports demonstrate that WHODAS can be collected in trials, but they do not establish a common treatment-response threshold. Future trials should prespecify the WHODAS form, outcome status, scoring, time point, and clinically interpretable change before responsiveness can be compared across interventions.

In medico-legal settings, the transferable implication is conceptual rather than jurisdiction-specific: an ICF-aligned patient report may complement impairment-based assessment by documenting activity and participation. However, the reviewed studies did not test eligibility algorithms, inter-rater consistency in benefit determination, or legal outcomes. WHODAS scores should, therefore, be clinically contextualized and must not be treated as direct proxies for organic impairment or entitlement.

This distinction is relevant across jurisdictions because administrative systems differ in statutory definitions, thresholds, adjudication procedures, and the weight assigned to self-report. The evidence synthesized here addresses measurement properties and research associations in adults with MS; it does not compare national assessment systems. Translation into policy would require prospective implementation studies that examine training, missing data, response bias, cultural adaptation, reproducibility across assessors, and the incremental contribution of WHODAS beyond clinical documentation. Equity analyses would also be necessary to determine whether environmental disadvantage or limited access to work and support influences scores or decisions. Until such evidence is available, WHODAS can inform a multidimensional description but cannot, on the basis of this review, be assigned a universal legal meaning.

The contribution of this review is to distinguish these applications and their evidential limits within a common measurement framework, rather than to infer that every WHODAS use provides the same information.

This distinction also improves interpretation of negative findings. A null secondary WHODAS result in a trial designed for falls does not invalidate the instrument, just as a strong cross-sectional correlation does not establish responsiveness or prediction. Evidence must be matched to the question, analysis population, form, score, and time point from which it arose. Maintaining such alignment is especially important when a generic patient-reported measure is used across research programs with very different objectives.

### 4.2. Methodological Implications, Limitations, and Future Directions

WHODAS should be interpreted as complementary to EDSS and MS-specific measures. EDSS remains necessary for neurological staging; WHODAS describes patient-reported activity and participation across life domains. A high score may reflect mobility impairment, fatigue, mood, cognition, pain, participation, work instability, or environmental barriers, and cannot be assumed to represent neurological progression.

Complementarity also has practical implications for study design. When the objective is neurological progression, WHODAS may add the patient perspective but should not substitute for validated clinical or performance measures. When the objective is lived functioning, EDSS alone may be insufficient, but an MS-specific symptom or quality-of-life measure may still be needed to identify the source of disability. Domain scores can improve interpretability only when their measurement properties and scoring are adequate in the population studied. The choice between 12-, 32-, and 36-item forms should, therefore, follow the level of inference, respondent burden, work-item applicability, and need for individual versus group interpretation.

Several limitations reduce confidence. Designs, settings, samples, WHODAS forms, scoring methods, outcome roles, comparators, and effect metrics varied substantially. Most clinical studies were cross-sectional; some samples were small; self-report was common; and domain reporting was incomplete. WHODAS was peripheral in twelve reports, which limited instrument-specific interpretation. Eight TONiC-MS publications and three additional overlap clusters meant that report counts overstated the amount of independent evidence.

Additional limitations concern review scope and reporting. Restriction to English-language peer-reviewed articles may have excluded null results, local adaptations, dissertations, or implementation reports. The search archive did not retain platform-generated command-line histories, so reproducibility is limited to the verified Boolean expression and interface fields. Some source reports did not state the WHODAS form, recall period, or scoring algorithm, and one validation article reported analysis samples inconsistently across sections. These items were coded as not reported rather than resolved by assumption.

The formal pooling audit found no pair or group of independent reports with a common construct, compatible WHODAS implementation, comparable time point, and common estimand. Rescaling scores would not make correlations, regression coefficients, treatment contrasts, and trajectory parameters equivalent. Narrative synthesis was, therefore, methodologically preferable. Publication and language bias are also possible because gray literature and non-English reports were excluded. Certainty was moderate only for measurement properties, low for quality-of-life and clinical-severity associations and longitudinal evidence, and low to very low for psychological, participation/employment, and intervention findings.

The decision not to pool should not be interpreted as an absence of quantitative evidence. Source-specific coefficients, confidence intervals, model fit, and group contrasts are retained in Supplementary Tables S13 and S16. The audit instead protects against a misleading summary estimate. A standardized mean difference, for example, would still combine different interventions and outcome roles; a pooled correlation would combine different constructs; and a count-based synthesis would over-weight TONiC-MS. The narrative approach preserves these distinctions while allowing consistency and uncertainty to be assessed transparently.

The review also cannot determine which WHODAS form is optimal for every clinical circumstance. The 12-item form reduces respondent burden but has weaker support for individual monitoring. The 32-item Rasch score is well suited to comparisons that should not depend on employment, yet it requires validated transformations. The 36-item form offers fuller domain coverage but includes work-related items that may be structurally missing for some participants. Selection should be driven by the planned inference, not by convenience alone. Direct head-to-head comparisons, independent responsiveness studies, and interpretable change thresholds remain priorities before routine individual-level monitoring can be recommended.

Future studies should report WHODAS form, administration, recall period, scoring, domain treatment, and outcome status. Longitudinal studies should prespecify whether WHODAS is an outcome, predictor, trajectory variable, or covariate; trials should report baseline and follow-up distributions and between-group estimates. Independent replication and predefined interpretability thresholds are required before prognostic, implementation, or benefit-determination claims can be supported.

For measurement research, priority should be given to independent confirmation of dimensionality and responsiveness, direct comparison of forms, and estimation of minimal important change in defined MS populations. For clinical and occupational research, analyses should distinguish neurological impairment from patient-reported functioning and specify confounders prospectively. For trials, WHODAS should be prespecified and powered when it is intended to support treatment claims. For service or medico-legal implementation, dedicated studies must evaluate reliability, decision consequences, fairness, and incremental information beyond existing clinical assessment.

## 5. Conclusions

WHODAS 2.0 has been used across multiple areas of MS research, but the evidential strength differs by purpose. Moderate-certainty evidence supports measurement properties of total scores, with important differences among 12-, 32-, and 36-item forms. Associations with clinical severity and quality of life are of low certainty, while psychological, participation, employment, and intervention findings are of low or very low certainty.

Accordingly, WHODAS may complement EDSS by describing patient-reported functioning beyond neurological impairment, but current evidence does not establish general clinical utility, prognostic performance, treatment responsiveness, or validity for benefit determination. Peripheral WHODAS use and overlapping cohorts further limit the interpretation of report counts.

Standardized form selection, scoring, domain reporting, prespecified outcome status, and independent longitudinal replication are needed. Until then, conclusions should remain specific to the measurement property, association, or intervention context evaluated in each study. Transparent reporting of these elements will allow future reviewers to judge when independent evidence is sufficiently comparable for a defensible quantitative synthesis.

This calibrated interpretation preserves the value of a broad, ICF-aligned patient report while preventing measurement evidence from being extended to decisions that the current studies did not evaluate.

## Figures and Tables

**Table 1:** Key characteristics and analytic role of WHODAS in the 28 included reports.

Study [Ref.]	Country	Design	MS Sample	WHODAS Form/Scoring	Analytic Role
Akhbardeh et al., 2020 [32]	USA	Cross-sectional sensor-validation	117 MS; 30 controls	Form not specified; total score	Secondary external disability criterion
Block et al., 2022 [33]	USA	Prospective remote-monitoring cohort	94	Form not specified; total score	Secondary predictor within a multimodal model
Curatoli et al., 2025 [34]	Italy	Cross-sectional	151	12-item; 0–100	Primary perceived-disability outcome
Gandy et al., 2025 [35]	Australia	Pooled predictor analysis	222	12-item	Primary disability-response criterion
Gandy et al., 2026 [36]	Australia	Randomized controlled trial	133	12-item	Primary disability outcome
Garin et al., 2010 [37]	Multi-country Europe	Longitudinal psychometric validation	1119 overall; 100 MS	36-item; 0–100	Measurement target; MS subgroup
Hoang et al., 2024 [38]	Australia	Multi-center randomized controlled trial	461 randomized; 453 primary analysis	Form not specified	Secondary outcome
Infortuna et al., 2025 [39]	Italy	Cross-sectional	105	36-item; IRT 0–100	Primary disability outcome
Magistrale et al., 2015a [40]	Italy	Cross-sectional clinical correlates	61 MS; 61 controls	36-item	Primary self-rated disability outcome
Magistrale et al., 2015b [41]	Italy	Psychometric validation	136	36-/32-item; classical and Rasch	Measurement target
Parker et al., 2025 [42]	UK	Longitudinal cohort	5210; 8603 surveys	32-item Rasch	Primary longitudinal disability outcome
Patten et al., 2007 [43]	Canada	Non-randomized intervention	83; full WHODAS in 52	WHO-DAS II; form incompletely reported	Secondary functional outcome
Pokryszko-Dragan et al., 2020 [44]	Poland	Cross-sectional	201	Form not specified; total/domains	Primary participation/disability outcome
Raggi et al., 2015 [45]	Italy	Instrument development/validation	181 employed	36-item; 0–100	Secondary convergent-validity comparator
Robinson et al., 2015 [46]	UK	Three-arm randomized controlled trial	56 randomized	12-item; 0–48	Secondary rehabilitation outcome
Rothman et al., 2023 [47]	UK	Cross-sectional psychosocial analysis	5059	32-item Rasch	Supportive functioning construct
Rothman et al., 2025 [48]	UK	Structural equation modeling	5266	32-item Rasch	Functioning outcome
Salehi et al., 2016 [49]	Iran	Cross-sectional	101	36-item	Primary disability correlate
Salehi et al., 2020 [50]	Iran	Cross-cultural psychometric validation	Not consistently reported across analyses	Persian 36-item	Measurement target
Schiavolin et al., 2016 [51]	Italy	Cross-sectional cut-off validation	180	36-item summary score	Secondary severity anchor
Slavkovic et al., 2019 [52]	Serbia	Cross-sectional	108	36-item	Primary current-functioning outcome
Vister et al., 2017 [53]	Australia	Prospective falls cohort; baseline association	210	12-item	Secondary functioning outcome
Wu et al., 2020 [54]	USA	Psychometric validation	256	12-item	Measurement target
Young et al., 2023 [55]	UK	Rasch psychometric validation	5809	36-/32-/12-item Rasch	Measurement target
Young et al., 2024a [56]	UK	Cross-sectional depression analysis	5627 with WHODAS data	32-item Rasch	Secondary clinical correlate
Young et al., 2024b [57]	UK	Longitudinal relapse-trajectory analysis	3885	32-item Rasch	Secondary disability-profile variable
Young et al., 2025 [58]	UK	Longitudinal inception/decade cohorts	813 inception; 679 decade	32-item Rasch	Primary disability-trajectory outcome
Young et al., 2026 [59]	UK	Longitudinal employment analysis	1865	32-item Rasch plus cognition domain	Predictor of employment transitions

Abbreviations: IRT = item response theory; MS = multiple sclerosis; WHODAS = World Health Organization Disability Assessment Schedule.

**Table 2:** Review-defined evidence streams, WHODAS roles, certainty, and principal limitations.

Evidence Stream	Reports (*n*)	Typical WHODAS Role/Forms	Principal Signal	Certainty	Main Limitations
Measurement properties	5	WHODAS 36/32/12 as the measurement target	Reliability, dimensionality, construct validity, Rasch scoring	Moderate	Version/scoring heterogeneity; one mixed-disease sample
Clinical associations	8	Usually central outcome or correlate	QoL, EDSS, fatigue, mood, cognition, motor function	Low to very low	Predominantly cross-sectional; reverse causality/confounding
Participation/employment	3	Comparator, anchor, or predictor	Social participation, job difficulty, employment transitions	Low to very low	Related occupational samples and TONiC overlap
Intervention/response	5	Primary in 2; secondary/contextual in 3	CBT, falls prevention, balance training, depression management	Very low to low	Different interventions, estimands, forms, and time points
Digital/remote monitoring	2	Secondary external criterion/predictor	Sensor-derived motor dysfunction and falls monitoring	Very low	Small stream; WHODAS not the sole analytic focus
Longitudinal/psychosocial	5	Outcome, profile variable, or mediator	Progression, relapse/diagnosis trajectories, psychosocial pathways	Low	Predominantly overlapping TONiC-MS reports

Abbreviations: CBT = cognitive behavioral therapy; QoL = quality of life; TONiC-MS = Trajectories of Outcome in Neurological Conditions in Multiple Sclerosis; WHODAS = World Health Organization Disability Assessment Schedule.

## Data Availability

No new datasets were generated or analyzed for this review. All data supporting the findings of this systematic review were extracted from published articles. The search strategy, eligibility framework, PRISMA flow counts, risk-of-bias approach, GRADE framework, and narrative synthesis procedures are provided in the manuscript and Supplementary Material S1.

## Supplementary Materials

The following supporting information can be downloaded at: https://www.mdpi.com/article/10.3390/medsci14050542/s1, Supplementary Material S1: Table S1: PRISMA 2020 and PRISMA-S reporting matrix; Table S2: PCC eligibility framework; Table S3: (A): Archived executed database strategy, field selection, dates, limits, and counts; (B): Exact database-specific search strings; Table S4: Study-selection workflow; Table S5: Full-text exclusion reasons; Table S6: Data-extraction fields; Table S7: Critical-appraisal tool allocation by design; Table S8: (A): Study-level design-specific appraisal allocation and synthesis-relevant concern; (B): Cross-report domain matrix corresponding to Figure 3; Table S9: Certainty-assessment framework; Table S10: SWiM grouping and synthesis rules; Table S11: PRISMA 2020 flow counts; Table S12: (A): Outcome-stream certainty summary; (B): Property-specific certainty within measurement evidence; Table S13: Detailed characteristics of included reports; Table S14: WHODAS implementation and analytic role; Table S15: Cross-stream meta-analysis feasibility audit; Table S16: Report-level quantitative findings, effect-size signals, and synthesis interpretation. Supplementary Material S2: PRISMA 2020 for Abstracts Checklist; Supplementary Material S3: PRISMA 2020 Checklist.
