# Supplementary material for: The Measurement Properties and Applications of WHODAS 2.0 in Multiple Sclerosis: A Systematic Review
Source: Med Sci (Basel). 2026 Sep 2;14(5):542. doi: 10.3390/medsci14050542 (PMC13609614; doi:10.3390/medsci14050542)
Supplement: Supplementary file 1 [file medsci-14-00542-s001.zip › Supplementary Materials S1.pdf]

Supplementary Material S1. Methodological and study-level tables

The Measurement Properties and Applications of WHODAS 2.0 in Multiple Sclerosis: A Systematic Review

This supplement reports the verified methodological record, design-specific and cross-report appraisal, property-specific certainty, study-level WHODAS implementation, cohort-overlap safeguards, meta-analysis feasibility audit, and report-level quantitative findings.

Supplementary Table S1. PRISMA 2020 and PRISMA-S reporting matrix

Domain	Operational decision	Location
Protocol	PROSPERO CRD420261403510; registered 23 May 2026.	Methods 2.1
Framework	PCC framework with six review-defined evidence streams.	Methods 2.2
Sources	Five databases searched from inception to 25 May 2026.	Methods 2.3; Tables S3A–S3B
Selection	Duplicate independent screening with adjudication.	Methods 2.4; Figure 1
Extraction	Duplicate extraction including WHODAS role and cohort family.	Methods 2.5
Appraisal	RoB 2, ROBINS-I, COSMIN, JBI, or QUIPS according to design.	Methods 2.6; Tables S7-S8
Certainty	Outcome-/stream-specific GRADE with COSMIN integration.	Methods 2.7; Table S12
Synthesis	SWiM plus formal meta-analysis feasibility audit.	Methods 2.8; Table S15

Supplementary Table S2. PCC eligibility framework

PCC element	Inclusion	Exclusion/interpretive rule
Population	Adults with physician-diagnosed MS; any phenotype, severity, treatment, or setting. Mixed samples required extractable MS data.	Pediatric-only, non-MS, or non-extractable mixed samples.
Concept	WHODAS 2.0/WHO-DAS II as measurement target, outcome, predictor, correlate, secondary measure, or disability descriptor; 12/32/36-item forms and reported scoring.	No empirical WHODAS result or adjacent disability instrument only.
Context	Clinical, community, psychometric, occupational, rehabilitation, digital, or longitudinal research.	No context restriction; context informed synthesis stream.
Evidence type	Original peer-reviewed English-language full articles.	Reviews, protocols without results, conference material, theses, grey literature, or non-English reports.

Supplementary Table S3A. Archived executed database strategy, field selection, dates, limits, and counts

Database	Field selection	Verbatim Boolean expression	Search date	Limits/operators	Records
PubMed/MEDLINE	All fields (interface selection)	("multiple sclerosis" OR "relapsing-remitting multiple sclerosis" OR "secondary progressive multiple sclerosis" OR "primary progressive multiple sclerosis") AND ("WHODAS" OR "WHODAS 2.0" OR "WHODAS-II" OR "WHODAS II" OR "WHO-DAS" OR "WHO-DAS II" OR "WHO DAS" OR "WHO DAS II" OR "WHODAS-12" OR "WHODAS 12" OR "WHODAS-32" OR "WHODAS 32" OR "WHODAS-36" OR "WHODAS 36" OR "World Health Organization Disability Assessment Schedule" OR "World Health Organisation Disability Assessment Schedule" OR "WHO Disability Assessment Schedule" OR "WHO Disability Assessment Schedule 2.0" OR "Disability Assessment Schedule II")	25 May 2026	Inception; no date/method filter; English applied at eligibility; no MeSH/proximity	27

Database	Field selection	Verbatim Boolean expression	Search date	Limits/operators	Records
Embase	All fields (interface selection) – Query Builder	("multiple sclerosis" OR "relapsing-remitting multiple sclerosis" OR "secondary progressive multiple sclerosis" OR "primary progressive multiple sclerosis") AND ("WHODAS" OR "WHODAS 2.0" OR "WHODAS-II" OR "WHODAS II" OR "WHO-DAS" OR "WHO-DAS II" OR "WHO DAS" OR "WHO DAS II" OR "WHODAS-12" OR "WHODAS 12" OR "WHODAS-32" OR "WHODAS 32" OR "WHODAS-36" OR "WHODAS 36" OR "World Health Organization Disability Assessment Schedule" OR "World Health Organisation Disability Assessment Schedule" OR "WHO Disability Assessment Schedule" OR "WHO Disability Assessment Schedule 2.0" OR "Disability Assessment Schedule II")	25 May 2026	Inception; no date/method filter; English applied at eligibility; no Emtree/proximity	59
Scopus	All fields (interface selection) – Query Builder – Advanced search.	("multiple sclerosis" OR "relapsing-remitting multiple sclerosis" OR "secondary progressive multiple sclerosis" OR "primary progressive multiple sclerosis") AND ("WHODAS" OR "WHODAS 2.0" OR "WHODAS-II" OR "WHODAS II" OR "WHO-DAS" OR "WHO-DAS II" OR "WHO DAS" OR "WHO DAS II" OR "WHODAS-12" OR "WHODAS 12" OR "WHODAS-32" OR "WHODAS 32" OR "WHODAS-36" OR "WHODAS 36" OR "World Health Organization Disability Assessment Schedule" OR "World Health Organisation Disability Assessment Schedule" OR "WHO Disability Assessment Schedule" OR "WHO Disability Assessment Schedule 2.0" OR "Disability Assessment Schedule II")	25 May 2026	Inception; no date/method filter; English applied at eligibility; no proximity	548
Web of Science	Topic/all-fields interface selection – Advanced Search	("multiple sclerosis" OR "relapsing-remitting multiple sclerosis" OR "secondary progressive multiple sclerosis" OR "primary progressive multiple sclerosis") AND ("WHODAS" OR "WHODAS 2.0" OR "WHODAS-II" OR "WHODAS II" OR "WHO-DAS" OR "WHO-DAS II" OR "WHO DAS" OR "WHO DAS II" OR "WHODAS-12" OR "WHODAS 12" OR "WHODAS-32" OR "WHODAS 32" OR "WHODAS-36" OR "WHODAS 36" OR "World Health Organization Disability Assessment Schedule" OR "World Health Organisation Disability Assessment Schedule" OR "WHO Disability Assessment Schedule" OR "WHO Disability Assessment Schedule 2.0" OR "Disability Assessment Schedule II")	25 May 2026	Inception; no date/method filter; English applied at eligibility; no proximity	28
Cochrane Library	All text/interface selection	("multiple sclerosis" OR "relapsing-remitting multiple sclerosis" OR "secondary progressive multiple sclerosis" OR "primary progressive multiple sclerosis") AND ("WHODAS" OR "WHODAS 2.0" OR "WHODAS-II" OR "WHODAS II" OR "WHO-DAS" OR "WHO-DAS II" OR "WHO DAS" OR "WHO DAS II" OR "WHODAS-12" OR "WHODAS 12" OR "WHODAS-32" OR "WHODAS 32" OR "WHODAS-36" OR "WHODAS 36" OR "World Health Organization Disability Assessment Schedule" OR "World Health Organisation Disability Assessment Schedule" OR "WHO Disability Assessment Schedule" OR "WHO Disability Assessment Schedule 2.0" OR "Disability Assessment Schedule II")	25 May 2026	Inception; no date/method filter; English applied at eligibility; no proximity	15

Search-strategy note: Supplementary Table S3A reports the database, field selection, search date, limits, operators, and retrieved count. Supplementary Table S3B reproduces the exact search strings applied in each database. Wording, quotation marks, Boolean operators, and database-specific syntax are reported verbatim.

Supplementary Table S3B. Exact database-specific search strings

Database	Exact database search string (verbatim)	Status
PubMed/MEDLINE	("multiple sclerosis" OR "relapsing-remitting multiple sclerosis" OR "secondary progressive multiple sclerosis" OR "primary progressive multiple sclerosis") AND ("WHODAS" OR "WHODAS 2.0" OR "WHODAS-II" OR "WHODAS II" OR "WHO-DAS" OR "WHO-DAS II" OR "WHO DAS" OR "WHO DAS II" OR "WHODAS-12" OR "WHODAS 12" OR "WHODAS-32" OR "WHODAS 32" OR "WHODAS-36" OR "WHODAS 36" OR "World Health Organization Disability Assessment Schedule" OR "World Health Organisation Disability Assessment Schedule" OR "WHO Disability Assessment Schedule" OR "WHO Disability Assessment Schedule 2.0" OR "Disability Assessment Schedule II")	Exact search string
Embase	('multiple sclerosis' OR 'relapsing-remitting multiple sclerosis' OR 'secondary progressive multiple sclerosis' OR 'primary progressive multiple sclerosis') AND ('whodas' OR 'whodas 2.0' OR 'whodas-ii' OR 'whodas ii' OR 'who-das' OR 'who-das ii' OR 'who das' OR 'who das ii' OR 'whodas-12' OR 'whodas 12' OR 'whodas-32' OR 'whodas 32' OR 'whodas-36' OR 'whodas 36' OR 'world health organization disability assessment schedule' OR 'world health organisation disability assessment schedule' OR 'who disability assessment schedule' OR 'who disability assessment schedule 2.0' OR 'disability assessment schedule ii')	Exact search string
Scopus	("multiple sclerosis" OR "relapsing-remitting multiple sclerosis" OR "secondary progressive multiple sclerosis" OR "primary progressive multiple sclerosis") AND ("WHODAS" OR "WHODAS 2.0" OR "WHODAS-II" OR "WHODAS II" OR "WHO-DAS" OR "WHO-DAS II" OR "WHO DAS" OR "WHO DAS II" OR "WHODAS-12" OR "WHODAS 12" OR "WHODAS-32" OR "WHODAS 32" OR "WHODAS-36" OR "WHODAS 36" OR "World Health Organization Disability Assessment Schedule" OR "World Health Organisation Disability Assessment Schedule" OR "WHO Disability Assessment Schedule" OR "WHO Disability Assessment Schedule 2.0" OR "Disability Assessment Schedule II")	Exact search string

Database	Exact database search string (verbatim)	Status
Web of Science Core Collection	("multiple sclerosis" OR "relapsing-remitting multiple sclerosis" OR "secondary progressive multiple sclerosis" OR "primary progressive multiple sclerosis") AND ("WHODAS" OR "WHODAS 2.0" OR "WHODAS-II" OR "WHODAS II" OR "WHO-DAS" OR "WHO-DAS II" OR "WHO DAS" OR "WHO DAS II" OR "WHODAS-12" OR "WHODAS 12" OR "WHODAS-32" OR "WHODAS 32" OR "WHODAS-36" OR "WHODAS 36" OR "World Health Organization Disability Assessment Schedule" OR "World Health Organisation Disability Assessment Schedule" OR "WHO Disability Assessment Schedule" OR "WHO Disability Assessment Schedule 2.0" OR "Disability Assessment Schedule II")	Exact search string
Cochrane Library	("multiple sclerosis" OR "relapsing-remitting multiple sclerosis" OR "secondary progressive multiple sclerosis" OR "primary progressive multiple sclerosis") AND ("WHODAS" OR "WHODAS 2.0" OR "WHODAS-II" OR "WHODAS II" OR "WHO-DAS" OR "WHO-DAS II" OR "WHO DAS" OR "WHO DAS II" OR "WHODAS-12" OR "WHODAS 12" OR "WHODAS-32" OR "WHODAS 32" OR "WHODAS-36" OR "WHODAS 36" OR "World Health Organization Disability Assessment Schedule" OR "World Health Organisation Disability Assessment Schedule" OR "WHO Disability Assessment Schedule" OR "WHO Disability Assessment Schedule 2.0" OR "Disability Assessment Schedule II")	Exact search string

Supplementary Table S4. Study-selection workflow

Step	Responsibility	Decision rule
Deduplication	Electronic deduplication plus manual checking	Title, authors, year, DOI/PMID, journal, and indexing variants.
Calibration	A.R. and M.G.M.	Pilot records discussed before formal screening.
Title/abstract	A.R. and M.G.M. independently	Either-reviewer inclusion advanced the record.
Full text	A.R. and M.G.M. independently	Mutually exclusive exclusion reason; R.S.C. adjudication.
Overlap	Compared authors, sites, dates, sample descriptions, and programme identifiers	Companion reports retained only for distinct WHODAS contributions.

Supplementary Table S5. Full-text exclusion reasons

Reason	n
No extractable WHODAS data	6
Wrong population/non-extractable MS subgroup	4
Ineligible publication type	4
Duplicate/overlapping report without distinct contribution	3
Adjacent disability instrument	2
Insufficient empirical data	1
Total full-text exclusions	20

Supplementary Table S6. Data-extraction fields

Domain	Fields
Identification/design	Author, year, country, setting, design, recruitment, funding/conflicts.
Participants	MS sample, phenotype, age, sex, disease duration, EDSS, treatment.
WHODAS	Form, administration, recall, scoring, domains, timing, outcome status, analytic role.
Results	Descriptive scores, associations, group/change estimates, precision, covariates.
Measurement properties	Reliability, validity, dimensionality, responsiveness, interpretability.
Overlap	Programme/cohort family, suspected shared participants, independent-count safeguard.
Meta-analysis audit	Construct, form, metric, time point, independence, transformability.

Critical appraisal was performed independently by A.R. and M.G.M.; unresolved disagreements were adjudicated by R.S.C.

Supplementary Table S7. Critical-appraisal tool allocation by design

Design/report type	Tool	Reports (n)	Primary focus
Randomized controlled trials	RoB 2	3	Randomization, deviations, missing outcomes, measurement, selective reporting.
Non-randomized intervention	ROBINS-I	1	Confounding, selection, classification, deviations, missingness, measurement, reporting.
Measurement-property studies	COSMIN	5	Design requirements for reliability, validity, structure, responsiveness, and interpretability.
Prognostic/trajectory analyses	QUIPS	6	Participation, attrition, prognostic-factor measurement, outcome measurement, confounding, analysis/reporting.
Analytical cross-sectional studies	JBI	13	Eligibility, measurement validity, confounding, outcome measurement, and analysis.

Supplementary Table S8A. Study-level design-specific appraisal allocation and synthesis-relevant concern

Study	Tool	Principal concern relevant to synthesis
Akhbardeh et al. 2020 [32]	JBI analytical cross-sectional checklist	Convenience/clinical sample; cross-sectional association.
Block et al. 2022 [33]	QUIPS	Attrition/model selection; WHODAS one of several predictors.
Curatoli et al. 2025 [34]	JBI analytical cross-sectional checklist	Cross-sectional temporal ambiguity and residual confounding.
Gandy et al. 2025 [35]	QUIPS	Secondary pooled predictor analysis; overlap and modest events-per-parameter.
Gandy et al. 2026 [36]	RoB 2	Self-reported outcome and incomplete follow-up; some concerns.
Garin et al. 2010 [37]	COSMIN	Mixed-disease sample; MS subgroup indirectness.
Hoang et al. 2024 [38]	RoB 2	WHODAS secondary; incomplete outcome detail; some concerns.
Infortuna et al. 2025 [39]	JBI analytical cross-sectional checklist	Cross-sectional psychological measures and residual confounding.
Magistrale et al. 2015a [40]	JBI analytical cross-sectional checklist	Small cross-sectional sample; possible overlap.
Magistrale et al. 2015b [41]	COSMIN	Some subscales underperformed; overlap possible.
Parker et al. 2025 [42]	QUIPS	Attrition/missingness and programme overlap.
Patten et al. 2007 [43]	ROBINS-I	Non-randomized allocation and confounding; serious concern.
Pokryszko-Dragan et al. 2020 [44]	JBI analytical cross-sectional checklist	Cross-sectional reverse causality.
Raggi et al. 2015 [45]	JBI analytical cross-sectional checklist	Employed-only sample; probable overlap.
Robinson et al. 2015 [46]	RoB 2	Small complete-case trial; self-reported secondary endpoint; some concerns.
Rothman et al. 2023 [47]	JBI analytical cross-sectional checklist	Cross-sectional modelling; TONiC overlap.
Rothman et al. 2025 [48]	JBI analytical cross-sectional checklist	Cross-sectional SEM does not establish causal mediation; TONiC overlap.
Salehi et al. 2016 [49]	JBI analytical cross-sectional checklist	Small cross-sectional sample; small sample; no cohort overlap was verified.
Salehi et al. 2020 [50]	COSMIN	Missing/inconsistent sample reporting across analyses; factor-structure uncertainty.
Schiavolin et al. 2016 [51]	JBI analytical cross-sectional checklist	Cut-off derivation within a related occupational cohort.
Slavkovic et al. 2019 [52]	JBI analytical cross-sectional checklist	Cross-sectional model and limited covariate control.
Vister et al. 2017 [53]	JBI analytical cross-sectional checklist	WHODAS secondary; cross-sectional association within prospective falls follow-up.
Wu et al. 2020 [54]	COSMIN	Convenience sample and listwise deletion.
Young et al. 2023 [55]	COSMIN	Strong measurement design; TONiC anchor for companion reports.
Young et al. 2024a [56]	JBI analytical cross-sectional checklist	Cross-sectional depression association; TONiC overlap.
Young et al. 2024b [57]	QUIPS	Trajectory modelling and programme overlap.
Young et al. 2025 [58]	QUIPS	Attrition/trajectory classification and programme overlap.
Young et al. 2026 [59]	QUIPS	Employment selection, confounding, and programme overlap.

Supplementary Table S8B. Cross-report domain matrix corresponding to Figure 3

Study	D1	D2	D3	D4	D5	D6	D7	Overall
Akhbardeh et al. 2020	Serious	Moderate	Low	Moderate	Serious	Low	Moderate	Serious
Block et al. 2022	Serious	Moderate	Low	Moderate	Serious	Moderate	Moderate	Serious
Curatoli et al. 2025	Serious	Serious	Low	Moderate	Serious	Moderate	Moderate	Serious
Gandy et al. 2025	Serious	Moderate	Low	Moderate	Low	Moderate	Moderate	Serious
Garin et al. 2010	Moderate	Moderate	Low	Moderate	Low	Moderate	Moderate	Moderate
Infortuna et al. 2025	Serious	Serious	Low	Moderate	Serious	Moderate	Moderate	Serious
Magistrale et al. 2015a	Serious	Moderate	Low	Moderate	Low	Moderate	Moderate	Serious
Magistrale et al. 2015b	Moderate	Moderate	Low	Moderate	Low	Moderate	Moderate	Moderate
Parker et al. 2025	Serious	Moderate	Low	Moderate	Low	Moderate	Moderate	Serious
Patten et al. 2007	Serious	Serious	Modera te	Serious	Serious	Serious	Moderate	Serious
Pokryszko-Dragan et al. 2020	Serious	Moderate	Low	Moderate	Low	Moderate	Moderate	Serious
Raggi et al. 2015	Moderate	Moderate	Low	Moderate	Low	Moderate	Moderate	Moderate
Rothman et al. 2023	Serious	Moderate	Low	Moderate	Low	Moderate	Moderate	Serious
Rothman et al. 2025	Serious	Moderate	Low	Moderate	Low	Moderate	Moderate	Serious
Salehi et al. 2016	Serious	Moderate	Low	Moderate	Low	Moderate	Moderate	Serious
Salehi et al. 2020	Moderate	Moderate	Low	Moderate	Low	Moderate	Moderate	Moderate
Schiavolin et al. 2016	Moderate	Moderate	Low	Moderate	Low	Moderate	Moderate	Moderate
Slavkovic et al. 2019	Serious	Moderate	Low	Low	Low	Moderate	Moderate	Serious
Vister et al. 2017	Serious	Moderate	Low	Moderate	Low	Moderate	Moderate	Serious
Wu et al. 2020	Moderate	Moderate	Low	Moderate	Low	Moderate	Moderate	Moderate
Young et al. 2023	Moderate	Moderate	Low	Moderate	Low	Moderate	Moderate	Moderate
Young et al. 2024a	Serious	Moderate	Low	Moderate	Low	Moderate	Moderate	Serious
Young et al. 2024b	Serious	Moderate	Low	Moderate	Low	Moderate	Moderate	Serious
Young et al. 2025	Serious	Moderate	Low	Moderate	Low	Moderate	Moderate	Serious
Young et al. 2026	Serious	Moderate	Low	Moderate	Low	Moderate	Moderate	Serious

Domains: D1, confounding; D2, selection of participants; D3, classification of interventions or exposures; D4, deviations from intended interventions or exposures; D5, missing data; D6, outcome measurement; D7, selection of the reported result. This retained ROBINS-I-aligned matrix is descriptive only and did not replace the formal design-specific allocation in Tables S7-S8A.

Supplementary Table S9. Certainty-assessment framework

Evidence type	Approach	Safeguard
Measurement properties	COSMIN methodological quality plus consistency/precision/indirectness.	Do not combine reliability, validity, and responsiveness into one effect.
Associations	GRADE domains applied to confidence in direction/magnitude.	Observational starting point; reverse causality and confounding considered.
Interventions	GRADE domains applied to between-group WHODAS effects.	Outcome status, missingness, and indirectness considered.
Longitudinal/prognostic	GRADE/QUIPS integration.	Companion reports do not count as independent replication.

Supplementary Table S10. SWiM grouping and synthesis rules

Component	Operational rule
Grouping	Six review-defined streams; separate measurement, association, intervention, and trajectory estimands.
Priority	Adjusted over unadjusted; longitudinal over cross-sectional when the construct was comparable.
Role	Measurement target, central outcome/predictor, or secondary/supportive.
Overlap	Report retained for distinct outcome but not counted as independent replication.
Quantitative reporting	Source-reported estimates and uncertainty retained without artificial common scaling.
Causality	Observational results described as associations; trial claims restricted to tested contrasts.

Supplementary Table S11. PRISMA 2020 flow counts

PRISMA item	Count	Details
Records identified	677	PubMed 27; Web of Science 28; Cochrane 15; Embase 59; Scopus 548.
Duplicates removed	100	Before screening.
Records screened	577	Title/abstract.
Records excluded	529	Title 438; abstract 91.
Full texts sought/retrieved	48/48	No report unavailable.
Full-text exclusions	20	Reasons in Table S5.
Reports included	28	Narrative synthesis.
Quantitative pools	0	No feasible independent homogeneous pool.

Supplementary Table S12A. Outcome-stream certainty summary

Outcome stream	Evidence base	Certainty	Rationale
Measurement properties	5	Moderate	Consistent total-scale reliability/construct validity; forms/scoring differed; one mixed-disease study.
Quality of life	Multiple, partly overlapping	Low	Consistent direction but different QoL measures, metrics, and observational designs.
Clinical severity/motor-cognitive	Multiple	Low	Clinically coherent associations; cross-sectional confounding and heterogeneous predictors.
Fatigue/mood/cognition	Multiple	Low to very low	Reverse causality, shared self-report, different constructs, and overlap.
Participation/employment	4 principal reports	Low to very low	Different occupational constructs and overlapping cohorts.
Intervention/response	5	Very low to low	Few heterogeneous studies; WHODAS secondary/contextual in three.
Longitudinal trajectories	Predominantly TONiC-MS	Low	Large samples but overlapping reports, missingness, and different models.

Supplementary Table S12B. Property-specific certainty within measurement evidence

Measurement property	Evidence base	Certainty	Rationale
Total-score reliability	Five measurement reports; direct contribution varied by form	Moderate	Generally consistent reliability signals, with heterogeneity in form, scoring, and one mixed-disease sample.
Construct validity	Five measurement reports; direct contribution varied by comparison	Moderate	Convergent and known-groups findings were directionally coherent, but instruments and samples differed.
Structural validity/dimensionality	Subset of the five measurement reports	Low	Factor and Rasch structures differed by form, scoring approach, and subscale; cross-cultural comparability was incomplete.
Responsiveness and individual interpretability	Sparse, form-specific subset	Low	Few independent change estimates, no established minimal clinically important difference, and form-specific smallest-detectable-difference results.

Supplementary Table S13. Detailed characteristics of included reports

Study	Country	Design	MS sample	Cohort/overlap	Verified WHODAS-related finding
Akhbardeh et al. 2020 [32]	United States	Cross-sectional sensor-validation	117 MS; 30 controls	Independent	Finger- and foot-tapping sensor features each correlated with WHODAS (r_s=0.82).
Block et al. 2022 [33]	United States	Prospective remote-monitoring cohort	94	Independent	WHODAS was associated with subsequent falls (OR 1.16, 95% CI 1.08–1.25) but was not the sole predictor.
Curatoli et al. 2025 [34]	Italy	Cross-sectional	151	Independent	EDSS, state anxiety, and depression independently predicted WHODAS (adjusted R^2=0.705).
Gandy et al. 2025 [35]	Australia	Pooled predictor analysis	222	Gandy programme; overlaps intervention datasets	The response model showed modest discrimination (AUC=0.67).
Gandy et al. 2026 [36]	Australia	Randomized controlled trial	133	Gandy programme	Internet-delivered CBT produced a small post-treatment WHODAS effect (Hedges g=0.28).
Garin et al. 2010 [37]	Multicountry Europe	Longitudinal psychometric validation	1,119 overall; 100 MS	Independent mixed-disease cohort	Reliability and known-groups validity were supported; MS evidence was indirect within a mixed-disease sample.
Hoang et al. 2024 [38]	Australia	Multicentre randomized controlled trial	461 randomized; 453 primary analysis	Independent	The WHODAS between-group effect was −0.4 (95% CI −3.5 to 2.6; p=0.775).
Infortuna et al. 2025 [39]	Italy	Cross-sectional	105	Independent	Anxiety was associated with greater disability and hyperthymic temperament with lower disability.
Magistrale et al. 2015a [40]	Italy	Cross-sectional clinical-correlates	61 MS; 61 controls	Possible overlap with Magistrale validation	Fatigue contributed more than EDSS to total disability (f^2=1.19 vs 0.31).
Magistrale et al. 2015b [41]	Italy	Psychometric validation	136	Possible overlap with clinical-correlates report	Total-scale reliability was strong; several subscales had limitations.
Parker et al. 2025 [42]	United Kingdom	Longitudinal cohort	5,210; 8,603 surveys	TONiC-MS	Fatigue, comorbidity, and lower self-efficacy were associated with worse longitudinal disability.
Patten et al. 2007 [43]	Canada	Non-randomized intervention	83; full WHODAS in 52	Independent	Functional status did not clearly differ between disease-management and usual-care groups.
Pokryszko-Dragan et al. 2020 [44]	Poland	Cross-sectional	201	Independent	Depression and mobility complaints were associated with worse disability and participation.
Raggi et al. 2015 [45]	Italy	Instrument development/validation	181 employed	Italian MSQ-Job cohort; probable overlap	MSQ-Job correlated with WHODAS summary disability (r=0.761).
Robinson et al. 2015 [46]	United Kingdom	Three-arm randomized controlled trial	56 randomized	Independent	Adjusted WHODAS differences versus control were −10.2 for Wii and −8.7 for traditional balance training.
Rothman et al. 2023 [47]	United Kingdom	Cross-sectional psychosocial analysis	5,059	TONiC-MS	WHODAS characterized disability within locus-of-control analyses but was not the primary focus.

Rothman et al. 2025 [48]	United Kingdom	Structural equation modelling	5,266	TONiC-MS	Self-efficacy mediated associations between locus of control and functioning/quality of life.
Salehi et al. 2016 [49]	Iran	Cross-sectional	101	No overlap verified; same-country setting only	Total WHODAS correlated inversely with total MSQoL-54 (r=−0.71).
Salehi et al. 2020 [50]	Iran	Cross-cultural psychometric validation	Not consistently reported across analyses	No overlap verified; same-country setting only	The Persian version showed high test–retest reliability and construct validity.
Schiavolin et al. 2016 [51]	Italy	Cross-sectional cut-off validation	180	Italian MSQ-Job cohort; probable overlap	WHODAS/MSQoL-54 clusters anchored MSQ-Job cut-offs (AUC 0.845–0.859).
Slavkovic et al. 2019 [52]	Serbia	Cross-sectional	108	Independent	Motor and cognitive variables explained 43% of WHODAS variance.
Vister et al. 2017 [53]	Australia	Prospective falls cohort; baseline association	210	Independent	Fatigue correlated with WHODAS (r=0.57); low activity was associated with worse functioning.
Wu et al. 2020 [54]	United States	Psychometric validation	256	Independent	A two-factor structure and good internal consistency were reported.
Young et al. 2023 [55]	United Kingdom	Rasch psychometric validation	5,809	TONiC-MS anchor report	The 36- and 32-item forms supported individual/group use; the 12-item form supported group use.
Young et al. 2024a [56]	United Kingdom	Cross-sectional depression analysis	5,627 with WHODAS data	TONiC-MS	Mean WHODAS was 47.2 with depression versus 38.1 without depression.
Young et al. 2024b [57]	United Kingdom	Longitudinal relapse-trajectory analysis	3,885	TONiC-MS	WHODAS differed across relapse-trajectory groups; relapse was the primary analytic focus.
Young et al. 2025 [58]	United Kingdom	Longitudinal inception/decade cohorts	813 inception; 679 decade	TONiC-MS	Three early disability trajectories were identified; 23.5% followed a high-disability trajectory.
Young et al. 2026 [59]	United Kingdom	Longitudinal employment analysis	1,865	TONiC-MS	Working participants had lower WHODAS scores than medically retired participants (34.5 vs 46.7).

Supplementary Table S14. WHODAS implementation and analytic role

Study	Form/scoring	Analytic role	Evidence stream	Overlap safeguard
Akhbardeh et al. 2020 [32]	Form not specified; total score	Secondary external disability criterion	Digital/remote	Independent
Block et al. 2022 [33]	Form not specified; total score	Secondary predictor within a multimodal model	Digital/remote	Independent
Curatoli et al. 2025 [34]	12-item; 0–100	Primary perceived-disability outcome	Clinical association	Independent
Gandy et al. 2025 [35]	12-item	Primary disability-response criterion	Intervention/response	Gandy programme; overlaps intervention datasets
Gandy et al. 2026 [36]	12-item	Primary disability outcome	Intervention/response	Gandy programme
Garin et al. 2010 [37]	36-item; 0–100	Measurement target; MS subgroup	Measurement properties	Independent mixed-disease cohort
Hoang et al. 2024 [38]	Form not specified	Secondary outcome	Intervention/response	Independent
Infortuna et al. 2025 [39]	36-item; IRT 0–100	Primary disability outcome	Clinical association	Independent
Magistrale et al. 2015a [40]	36-item	Primary self-rated disability outcome	Clinical association	Possible overlap with Magistrale validation
Magistrale et al. 2015b [41]	36-/32-item; classical and Rasch	Measurement target	Measurement properties	Possible overlap with clinical-correlates report
Parker et al. 2025 [42]	32-item Rasch	Primary longitudinal disability outcome	Longitudinal/psychosocial	TONiC-MS
Patten et al. 2007 [43]	WHO-DAS II; form incompletely reported	Secondary functional outcome	Intervention/response	Independent
Pokryszko-Dragan et al. 2020 [44]	Form not specified; total/domains	Primary participation/disability outcome	Clinical association	Independent
Raggi et al. 2015 [45]	36-item; 0–100	Secondary convergent-validity comparator	Participation/employment	Italian MSQ-Job cohort; probable overlap
Robinson et al. 2015 [46]	12-item; 0–48	Secondary rehabilitation outcome	Intervention/response	Independent
Rothman et al. 2023 [47]	32-item Rasch	Supportive functioning construct	Longitudinal/psychosocial	TONiC-MS
Rothman et al. 2025 [48]	32-item Rasch	Functioning outcome	Longitudinal/psychosocial	TONiC-MS
Salehi et al. 2016 [49]	36-item	Primary disability correlate	Clinical association	No overlap verified; same-country setting only
Salehi et al. 2020 [50]	Persian 36-item	Measurement target	Measurement properties	No overlap verified; same-country setting only
Schiavolin et al. 2016 [51]	36-item summary score	Secondary severity anchor	Participation/employment	Italian MSQ-Job cohort; probable overlap
Slavkovic et al. 2019 [52]	36-item	Primary current-functioning outcome	Clinical association	Independent
Vister et al. 2017 [53]	12-item	Secondary functioning outcome	Clinical association	Independent
Wu et al. 2020 [54]	12-item	Measurement target	Measurement properties	Independent
Young et al. 2023 [55]	36-/32-/12-item Rasch	Measurement target	Measurement properties	TONiC-MS anchor report
Young et al. 2024a [56]	32-item Rasch	Secondary clinical correlate	Clinical association	TONiC-MS
Young et al. 2024b [57]	32-item Rasch	Secondary disability-profile variable	Longitudinal/psychosocial	TONiC-MS
Young et al. 2025 [58]	32-item Rasch	Primary disability-trajectory outcome	Longitudinal/psychosocial	TONiC-MS
Young et al. 2026 [59]	32-item Rasch plus cognition domain	Predictor of employment transitions	Participation/employment	TONiC-MS

Supplementary Table S15. Cross-stream meta-analysis feasibility audit

Evidence stream	Numerically informative reports	Pool feasible?	Reason pooling/transformation was not undertaken
Measurement properties	5	No	Reliability, factor/Rasch, validity, and responsiveness indices addressed different properties and forms; these are not one estimand.
Quality of life	6 reports with a WHODAS–QoL signal	No	Different QoL instruments and metrics (correlations, regression paths, group contrasts), with potential sample overlap.
Clinical/motor-cognitive associations	At least 8	No	Different predictors, covariate sets, domains, and cross-sectional estimands.
Psychological/fatigue associations	At least 7	No	Different constructs/scales; reciprocal relationships; several TONiC analyses were not independent.
Participation/employment	4	No	Different outcomes and two overlap clusters (MSQ-Job and TONiC-MS).
Intervention/response	5; 4 reported group/change results	No	Different interventions, comparators, WHODAS roles/forms, time points, and effect metrics.
Longitudinal trajectories	8 TONiC-related topical contributions plus 1 remote cohort	No	Most reports shared the TONiC programme; outcomes and models were non-equivalent.

Decision rule: pooling required at least two independent reports with the same construct, compatible WHODAS implementation, comparable time point, and a common estimand. No stream satisfied all conditions. Transforming scores to 0–100 would change the scale but would not harmonize the underlying estimand or remove cohort overlap. Counts in the 'numerically informative reports' column are cross-stream topical contributions and may exceed the mutually exclusive primary-stream totals shown in Figures 4-5; they are not independent-cohort counts.

Supplementary Table S16. Report-level quantitative findings, effect-size signals, and synthesis interpretation

Study [Ref.]	Clinical/measurement domain	Statistical approach and quantitative signal	Direction and interpretive contribution	Applicability and methodological considerations
Akhbardeh et al. [32]	Digital motor assessment	Spearman correlations between optimized multisensor textural features and WHODAS total score. Finger taps rs=0.82; foot taps rs=0.82; both p<0.0001.	Higher sensor-derived dysfunction tracked higher patient-reported disability. Supports convergent validity between WHODAS disability and sensor-derived upper- and lower-limb dysfunction, suggesting that patient-reported functional burden aligns with objective digital motor features.	Most applicable to criterion-association and digital-measure validation; cross-sectional design limits prognostic or responsiveness interpretation.
Block et al. [33]	Falls and remote monitoring	Random forest/stepwise prediction using remote step counts and patient-reported outcomes (PROs). WHODAS OR=1.16 (95% CI 1.08–1.25) for subsequent falls; WHODAS remained one component of a multimodal model.	Higher WHODAS disability was associated with greater fall risk. Positions WHODAS within a remote-monitoring disability profile that can complement gait-specific and wearable indicators of fall risk.	Most applicable to multimodal fall-risk monitoring; WHODAS was one patient-reported outcome within a broader prediction set, so findings should not be read as a WHODAS-specific falls model.
Curatoli et al. [34]	Perceived disability	Forward multivariable regression with WHODAS 2.0 12-item score as outcome. EDSS beta=7.190; state anxiety beta=0.265; BDI-II beta=0.835; adjusted R2=0.705; all p<=0.009.	Objective neurological disability and affective symptoms were independently associated with higher WHODAS disability. Shows that perceived disability on WHODAS integrates neurological severity with affective symptoms, supporting multidimensional disability assessment in MS rehabilitation.	Cross-sectional adjusted association; directionality between mood symptoms and perceived disability cannot be established.
Gandy et al. [35]	Clinical response to internet-delivered CBT	Logistic predictor models for >=25% disability response. Disability response in 82/203 participants (40%); model chi-square(4)=17.58, p=0.001; Nagelkerke R2=0.11; AUC=0.67.	Baseline profiles had limited discriminatory value for predicting WHODAS disability response. Demonstrates that WHODAS 2.0 can operationalize disability response in digital psychological-care datasets, although baseline predictors explained only modest variance.	Best applied to response-characterization rather than efficacy inference; derived from pooled intervention datasets within the Gandy programme.

Study [Ref.]	Clinical/measurement domain	Statistical approach and quantitative signal	Direction and interpretive contribution	Applicability and methodological considerations
Gandy et al. [36]	Internet-delivered CBT efficacy	RCT group-by-time analysis for WHODAS disability. Hedges g=0.28 for WHODAS disability at post-treatment; average disability reduction 14% in treatment versus 1% in control; p<0.05.	Small but statistically significant disability improvement in the intervention group. Provides randomized evidence that a digital CBT-based intervention can produce a small improvement in WHODAS disability alongside mood outcomes.	Most applicable to psychological intervention contexts; effect magnitude is small and should not be pooled with heterogeneous exercise or balance interventions without compatible metrics.
Garin et al. [37]	Psychometric validity	Reliability, factor analysis, known-groups validity and responsiveness across chronic diseases. Cronbach alpha 0.77-0.98; global WHODAS-2 mean 24.8; mild-severe effect sizes >0.7 for most conditions.	Higher disease severity corresponded to higher disability; psychometric reliability was strong. Provides broad generic-measurement support for WHODAS-2 reliability and known-groups validity, including an MS subgroup.	Indirect MS evidence from a mixed chronic-disease cohort; disease-specific MS interpretation should rely primarily on MS-only validation studies.
Hoang et al. [38]	Step exergame/falls prevention	Negative binomial regression for falls; secondary-outcome framework included psychosocial disability. WHODAS between-group effect −0.4 (95% CI −3.5 to 2.6), p=0.775; falls IRR=0.96 (95% CI 0.69–1.34).	No evidence of a WHODAS or falls benefit. Shows how WHODAS can be embedded as a secondary disability/functioning endpoint in a large falls-prevention rehabilitation trial.	Falls were the primary endpoint; WHODAS-specific treatment effects were not central to the primary report, so the study contributes contextual rehabilitation evidence.
Infortuna et al. [39]	Temperament, psychological symptoms and disability	Hierarchical regressions for WHODAS domains and total score. WHODAS total final model R2=0.379; hyperthymic beta=-0.257, p=0.010; anxiety beta=0.409, p=0.006.	Hyperthymic traits associated with less disability; anxiety associated with greater disability. Links WHODAS disability to temperament and psychological symptoms, extending WHODAS interpretation toward personal and affective correlates of functioning.	Cross-sectional design; temperament and anxiety associations should be treated as correlates rather than causal pathways.
Magistrale et al. [40]	Fatigue, EDSS and self-rated disability	Multivariable regression predicting WHODAS-II domains and total score. Total score: MFIS Cohen f2=1.19; EDSS f2=0.31; R2=0.601; adjusted R2=0.586.	Fatigue showed the largest adjusted association to self-rated disability; EDSS added physical-disability contribution. Clarifies that WHODAS self-rated disability in MS is strongly shaped by fatigue and not merely by EDSS impairment severity.	Clinical-correlates evidence from a modest sample; potential relationship with the Italian validation cohort should be considered in sample-size aggregation.
Magistrale et al. [41]	WHODAS-II psychometric validation	Cronbach alpha, ICC/validity and Rasch model analyses. Total scale alpha=0.93; Rasch PSI=0.83 for total scale; subscale alpha range 0.68-0.96.	Total WHODAS-II score showed good reliability and validity; some subscales had limitations. Provides MS-specific evidence that the WHODAS-II total score has strong internal consistency and Rasch reliability, while some subscales require cautious interpretation.	Measurement-property study; reliability and validity indices should be summarized separately from clinical association effect sizes.
Parker et al. [42]	Longitudinal disability progression	Multilevel models of Rasch-transformed WHODAS 2.0 disability estimates. +5.83 WHODAS points per 1 SD NFI-MS (95% CI 5.46-6.21); +1.41 per comorbidity (95% CI 1.17-1.65); -7.71 per 1 SD USE-MS (95% CI -8.11 to -7.30).	Fatigue and comorbidity increased disability; higher self-efficacy reduced disability. Provides large-scale longitudinal evidence that fatigue, comorbidity burden and self-efficacy are quantitatively associated with WHODAS disability progression.	TONiC-MS companion analysis; most applicable to longitudinal disability modelling rather than independent sample-count synthesis.
Patten et al. [43]	Depression disease management	Before-after/intervention group comparison with 6-month follow-up. Major depression at 6 months: 33.3% disease-management versus 55.2% treatment-as-usual; Fisher p=0.06; WHODAS II difference between groups was not observed.	Depression outcome improved directionally; functional status did not clearly differ. Places WHO-DAS II functional status within depression-care management in MS, showing that functional improvement was less evident than depressive-symptom change.	Non-randomized management study with WHODAS as secondary functional outcome; limited suitability for WHODAS responsiveness conclusions.
Pokryszko-Dragan et al. [44]	Social participation and disability	General linear models/correlations with WHODAS total and domains. 27.36% above 90th percentile versus population norms; BDI-WHODAS association beta=0.73, p<0.001; mobility symptom beta=0.26 for total WHODAS.	Depression and mobility complaints were associated with worse WHODAS disability/participation. Supports WHODAS as a participation-sensitive disability measure, with depression and mobility complaints strongly associated with worse functioning.	Cross-sectional participation study; observed associations may reflect reciprocal relationships between mood, mobility and social functioning.
Raggi et al. [45]	Work difficulties	Pearson correlations between MSQ-Job and WHODAS 2.0. MSQ-Job total with WHODAS summary disability r=0.761; work activities r=0.714; participation r=0.697; p<0.0007.	Greater work difficulty was strongly associated with broader disability and participation restrictions. Demonstrates strong convergence between WHODAS disability and MS-specific work-difficulty burden, supporting WHODAS relevance to occupational functioning.	Occupational validation evidence; interpreted together with Schiavolin et al. as complementary work-disability evidence from closely related Italian MSQ-Job datasets.
Robinson et al. [46]	Balance/exergaming rehabilitation	ANCOVA for post-intervention WHODAS 2.0, adjusted for baseline values and covariates. Adjusted WHODAS difference versus control: Wii −10.2 (95% CI −13.5 to −6.8); traditional balance −8.7 (−12.2 to −5.2).	Both exergaming and traditional balance training improved WHODAS versus control. Suggests that WHODAS 2.0 can detect short-term changes in activity and participation after balance-oriented rehabilitation.	Small trial, complete-case analysis, and secondary WHODAS outcome; magnitude may be unstable.
Rothman et al. [47]	Locus of control and psychosocial functioning	Rasch-transformed PROM fit and disability-stratified comparisons. WHODAS32 reliability=0.97 in calibration sample; LOC groups associated with QoL and self-efficacy (p<0.001).	WHODAS supported disability/functioning measurement within psychosocial modeling. Shows that WHODAS32 functions as a robust disability metric within psychosocial models linking locus of control, self-efficacy and quality of life.	TONiC-MS companion analysis; WHODAS supports the psychosocial framework but was not the sole primary measurement focus.

Study [Ref.]	Clinical/measurement domain	Statistical approach and quantitative signal	Direction and interpretive contribution	Applicability and methodological considerations
Rothman et al. [48]	Self-efficacy mediation	Structural equation modeling with functioning/disability outcome. Example CLOC-functioning validation model RMSEA=0.000, CFI=1.000, TLI=1.000; direct effects largely mediated by self-efficacy.	Self-efficacy mediated links between locus-of-control orientations and functioning/QoL. Uses WHODAS functioning within structural equation modelling to support self-efficacy as a mediator between locus-of-control orientations and functioning/quality of life.	TONiC-MS companion structural model analysis; findings support pathway plausibility but not causal identification.
Salehi et al. [49]	QoL-disability association	Pearson correlations and stepwise regression. Total WHODAS with total QoL r=-0.71; participation r=-0.70; regression R2=0.590; participation beta=-0.575; cognition beta=-0.277.	Higher WHODAS disability, especially participation and cognition, was associated with lower QoL. Provides direct evidence that WHODAS total disability, especially participation and cognition domains, is strongly associated with MS-specific quality of life.	Cross-sectional quality-of-life/disability study; no cohort overlap was verified; estimates support association, not causal direction.
Salehi et al. [50]	Persian WHODAS validation	Reliability, construct validity, EFA and CFA. ICC 0.82-0.99; all domain alpha >0.70; total WHODAS-MSQoL physical r=-0.744 and mental r=-0.655; SEM=0.31; MDC=0.86.	Persian WHODAS showed strong reliability and construct validity. Supports cross-cultural validity of the Persian WHODAS 2.0 in MS, with strong test-retest reliability and construct validity against MSQoL-54.	Psychometric and cultural-adaptation evidence; scoring considerations and missing item patterns should be considered when comparing total scores.
Schiavolin et al. [51]	Work-difficulty cutoff	K-means clusters based on WHODAS 2.0 and MSQoL-54; ROC analysis for MSQ-Job cutoffs. AUC=0.845 for MSQ-Job total; AUC=0.859 for count of MSQ-Job items scored >=3; Kendall tau=0.885.	WHODAS-anchored severity clusters supported MSQ-Job cut-off values. Shows that WHODAS-anchored disability and quality-of-life clusters can inform clinically meaningful MSQ-Job cut-off scores.	Occupational cut-off study; interpreted with Raggi et al. as related Italian MSQ-Job evidence rather than independent population-level evidence.
Slavkovic et al. [52]	Motor-cognitive functioning	Correlations and hierarchical regression predicting WHODAS 2.0 total score. EDSS-WHODAS r=0.34; ARCS r=-0.34; 9HPT r=0.48; 25FWT r=0.42; final model R2=0.43; Cohen f2=0.75.	Poorer cognition, worse upper-limb dexterity and worse walking performance aligned with worse WHODAS functioning. Supports WHODAS as an integrative current-functioning outcome linking neurological status, cognition, upper-limb dexterity and walking performance.	Independent cross-sectional rehabilitation study; EDSS alone did not explain functioning after cognitive and motor variables were considered.
Vister et al. [53]	Fatigue, activity and falls	Group comparisons and correlations. FSS-WHODAS r=0.57, p<0.001; high fatigue WHODAS 31.8 versus low fatigue 22.6; low walking activity WHODAS 30.4 versus high walking activity 25.9.	Fatigue and low walking activity were associated with worse WHODAS functioning and higher fall-risk context. Links WHODAS functioning with fatigue and walking activity in a prospective falls cohort, supporting its sensitivity to daily-life activity burden.	WHODAS was a broad life-functioning outcome rather than a fall-specific measure; prospective falls follow-up does not make WHODAS a standalone fall predictor.
Wu et al. [54]	12-item WHODAS psychometrics	Exploratory factor analysis, confirmatory factor analysis and concurrent validity. Two-factor model explained 60.50% variance; total alpha=0.90; self-care alpha=0.89; social/cognitive alpha=0.80; modified CFA CFI=0.961, RMSEA=0.075.	Two interpretable factors supported self-care and social/cognitive functioning dimensions. Provides MS-specific validation for the 12-item WHODAS structure, highlighting self-care and social/cognitive functioning dimensions relevant to vocational rehabilitation.	Community convenience sample; strongest contribution is measurement validity rather than prevalence or clinical prediction.
Young et al. [55]	WHODAS 2.0 validation and scoring	Rasch analysis, responsiveness, test-retest reliability and trajectory analysis. WHODAS-36 SDD=5.466; WHODAS-32 SDD=5.733; WHODAS-12 SDD=6.543. EDSS 0-4 versus 8-9.5 effect size=1.66 for WHODAS-32.	WHODAS-36/32 support individual use; WHODAS-12 supports group use only. Establishes the core MS-specific WHODAS 2.0 scoring framework, including interval transformation, responsiveness and individual-versus-group use recommendations.	TONiC-MS anchor measurement report; subsequent TONiC analyses should be interpreted as companion applications of this measurement framework.
Young et al. [56]	Depression and disability	t tests and logistic regression with depression status. WHODAS32 depressed 47.2 versus not depressed 38.1; effect size=0.882; logistic OR=1.020 per WHODAS32 point, p=0.012.	Depression was associated with substantially worse WHODAS disability. Shows a large disability difference by depression status and quantifies WHODAS32 as an independent correlate of depression in MS.	TONiC-MS companion analysis; findings apply to depression-disability association and should not be treated as an independent cohort from other TONiC reports.
Young et al. [57]	Relapse trajectories	Group-based trajectory modeling and profile comparisons. Inception trajectory WHODAS: 27.7 versus 36.0; trilogy groups 31.1, 35.6 and 42.1; all group comparisons p<0.05.	Higher relapse-trajectory burden aligned with worse WHODAS disability. Demonstrates that relapse-trajectory groups differ in WHODAS disability burden, linking patient-reported disability to longitudinal relapse patterns.	TONiC-MS companion relapse analysis; WHODAS contributed to trajectory profiling while relapse outcomes were the primary analytic focus.
Young et al. [58]	Diagnosis-period disability trajectories	Group-based trajectory modeling and CART. WHODAS32 trajectory groups: low 33.8%, medium 42.7%, high 23.5%; high-disability group baseline WHODAS 60.6; CART RHR 9.26 for poor health status in high-risk profile.	A sizable subgroup had high disability from diagnosis onward. Identifies distinct early-diagnosis WHODAS disability trajectories, showing that high disability may be present from the first year after diagnosis.	TONiC-MS companion inception/trajectory analysis; applicable to early-disease heterogeneity and service-planning interpretation.
Young et al. [59]	Employment transitions	Regression and CART for work disability/discontinuity. Medical-retirement model: WHODAS cognition OR=1.188 per point, p<0.001; WHODAS32 working mean 34.5 versus medically retired 46.7.	Higher cognitive-domain disability and global disability aligned with adverse employment outcomes. Quantifies the relationship between WHODAS cognition/global disability and adverse employment transitions, extending WHODAS relevance to work participation.	TONiC-MS companion employment analysis; strongest inference concerns employment-association modelling rather than independent sample aggregation.

Abbreviations: PROs, patient-reported outcomes. All other abbreviations are defined in the general abbreviation list below. Estimates are retained in their source-reported metric and are not treated as mutually poolable.

Abbreviations: 9HPT, Nine-Hole Peg Test; 25FWT, Timed 25-Foot Walk; ANCOVA, analysis of covariance; ARCS, Audio Recorded Cognitive Screen; AUC, area under the receiver operating characteristic curve; BDI-II, Beck Depression Inventory-II; CART, classification and regression tree; CBT, cognitive behavioural therapy; CFA, confirmatory factor analysis; CFI, comparative fit index; CI, confidence interval; CLOC, chance locus of control; COSMIN, COnsensus-based Standards for the selection of health Measurement INstruments; EDSS, Expanded Disability Status Scale; EFA, exploratory factor analysis; FSS, Fatigue Severity Scale; GRADE, Grading of Recommendations Assessment, Development and Evaluation; ICC, intraclass correlation coefficient; ICF, International Classification of Functioning, Disability and Health; IRR, incidence rate ratio; IRT, item response theory; JBI, Joanna Briggs Institute; LOC, locus of control; MDC, minimal detectable change; MFIS, Modified Fatigue Impact Scale; MS, multiple sclerosis; MSQ-Job, Multiple Sclerosis Questionnaire for Job Difficulties; MSQoL-54, Multiple Sclerosis Quality of Life-54; NFI-MS, Neurological Fatigue Index for Multiple Sclerosis; OR, odds ratio; PCC, Population-Concept-Context; PRISMA, Preferred Reporting Items for Systematic Reviews and Meta-Analyses; PROM, patient-reported outcome measure; PSI, Person Separation Index; QoL, quality of life; QUIPS, Quality In Prognosis Studies; RCT, randomized controlled trial; RHR, relative hazard ratio; RMSEA, root mean square error of approximation; RoB 2, revised Cochrane Risk of Bias tool; ROBINS-I, Risk Of Bias In Non-randomized Studies of Interventions; ROC, receiver operating characteristic; SDD, smallest detectable difference; SWiM, Synthesis Without Meta-analysis; TLI, Tucker-Lewis index; TONiC-MS, Trajectories of Outcome in Neurological Conditions in Multiple Sclerosis; USE-MS, Unidimensional Self-Efficacy Scale for Multiple Sclerosis; WHODAS, World Health Organization Disability Assessment Schedule.
